# DNA methylation is involved in the pathogenesis of osteoarthritis by regulating *CtBP* expression and CtBP-mediated signaling

**DOI:** 10.7150/ijbs.39945

**Published:** 2020-01-30

**Authors:** Xiangxiang Sun, Lin Xiao, Juan Chen, Xun Chen, Xinlin Chen, Shuxin Yao, Hui Li, Guanghui Zhao, Jianbing Ma

**Affiliations:** Department of Orthopaedics, Honghui Hospital, Xi'an Jiaotong University, Xi'an 710054, Shaanxi, China.

**Keywords:** DNA methylation, CtBP, p300, AP1, NLRP3, IL-1B.

## Abstract

Osteoarthritis (OA) is a common type of arthritis. Chronic inflammation is an important contributor to the pathogenesis of OA. The maturation and secretion of proinflammatory cytokines are controlled by inflammasomes, especially NLRP1 (NLR Family Pyrin Domain Containing 1) and NLRP3. In this study, we identified a transactivation mechanism of *NLRP3* mediated by CtBPs (C-terminal-binding proteins). We found that both the mRNA and protein levels of CtBPs were significantly increased in OA biopsies. Analyzing the profiles of differentially expressed genes in *CtBP*-knockdown and overexpression cells, we found that the expression of *NLRP3* was dependent on *CtBP* levels. By the knockdown or overexpression of transcription factors that potentially bind to the promoter of *NLRP3*, we found that only AP1 could specifically regulate the expression of NLRP3. Using immunoprecipitation (IP) and Co-IP assays, we found that AP1 formed a transcriptional complex with a histone acetyltransferase p300 and CtBPs. The knockdown of any member of this transcriptional complex resulted in a decrease in the expression of *NLRP3*. To explore the underlying mechanism of *CtBP* overexpression, we analyzed their promoters and found that they were abundant in CpG islands. Treatment with the DNA methylation inhibitor 5-aza-2′-deoxycytidine (AZA) or knockdown of *DNMT*s (DNA methyltransferases) resulted in the overexpression of *CtBP*s, while overexpression of *DNMT*s caused the reverse effects on *CtBP* expression. Collectively, our results suggest that the decreased DNA methylation levels in the promoters of *CtBP*s upregulate their expression. Increased CtBPs associated with p300 and AP1 to form a transcriptional complex and activate the expression of *NLRP3* and its downstream signaling, eventually aggravating the inflammatory response and leading to the pathogenesis of OA.

## Introduction

Osteoarthritis (OA) is a chronic disorder caused by damage or degeneration of cartilage in joints 1, 2. It is estimated that nearly 10%-15% of the population over 60 years old have different degrees of OA, which often leads to stiffness, pain, impaired movement and even severe disability 3. OA was originally considered as a noninflammatory arthritis because early findings observed fewer leukocytes in OA synovial fluid in comparison to those in other arthritis, such as rheumatoid arthritis (RA), reactive arthritis and septic arthritis 4. However, recent investigations have revealed that inflammation is strongly implicated in the pathogenesis of OA 4. Proinflammatory cytokines such as IL-1β (*Interleukin 1 beta) 5*, IL-6 6, and TNF-α (tumor necrosis factor-alpha) 7, chemokines such as CCL2 (chemokine ligand 2) and CCL20 8, 9, and other inflammatory mediators such as inflammasome members NLRP1 (NLR Family Pyrin Domain Containing) and *NLRP3* are elevated or activated in OA patients 10. Of these inflammatory cytokines, IL-1β has been well investigated, and it can induce the expression of multiple genes, such as *MMP-1* (Matrix Metallopeptidase 1), *MMP-3*, *MMP-8*, *MMP-13*, and C*OL1A2* (Type II collagen), in OA cartilages 11. Inflammasome NLRP3 can activate Caspase-1, which further cleaves pro-IL-1β to promote its maturation and secretion 12. In addition, multiple signaling pathways are activated in the pathogenesis of OA. For instance, the activation of the TLR4 (Toll-like receptor 4)/NF-κB (Nuclear Factor Kappa B) signaling pathway can regulate the expression of numerous cytokines, including IL-6, IL-8, IL-9 and IL-15 13. The activation of ROS (reactive oxygen species)-mediated signaling in cartilage can lead to chondrocyte damage and cell death 14. Wnt/β-catenin signaling is also activated and associated with OA progression and severity 15. Inhibition of Wnt/β-catenin signaling can decrease the progression of OA and improve its severity 15. A variety of these activated signaling pathways eventually affect gene expression in the nucleus by mediating transcription factors 16, 17.

Gene transcription is precisely controlled by transcriptional complexes, which commonly include transcription factors [e.g., NF-κB, AP-1 (Activator protein 1), CREB (cyclic AMP response element-binding protein) and STATs (signal transducers and activators of transcription)], coactivators [e.g., histone acetyltransferase p300 and CBP (CREB binding protein)] and corepressors [e.g., NcoR1 (Nuclear receptor corepressor 1) and CtBPs (*C-terminal-binding proteins*)] 18-21. The transcriptional complexes then promote or block the recruitment of RNA polymerase II to the promoters of specific genes 22. A number of transcription factors such as NF-κB, SP1 (Specificity protein 1), KLF4 (Kruppel like factor 4), JDP2 (Jun dimerization protein 2), and c-MYC have been found to participate in the regulation of genes involved in the pathogenesis of OA 23-26. Histone acetyltransferases (HATs) function to acetylate histone proteins, which impairs DNA-histone associations to make DNA more accessible by transcription factors, thereby inducing gene expression 27-29. Transcriptional corepressors like CtBPs mainly function to repress gene expression by directly interacting with HATs or HDACs (histone deacetylases) 30, 31. The human genome contains two CtBP members, CtBP1 and CtBP2, which share nearly 80% protein sequence identity 32. The current understanding is that CtBPs have unique roles in different biological processes. CtBP1 functions as an oncogene and is overexpressed in a variety of cancers 32. Overexpression of *CtBP1* can downregulate multiple tumor suppressors, such as *BRCA1* (Breast cancer susceptibility gene 1), *CDH1* (Cadherin 1), *Bax* (BCL2-associated X), *Bim* (Bcl-2 interacting mediator of cell death), and *PTEN* (phosphatase and tensin homolog) 32. CtBP2 is also overexpressed in some cancer types, such as gastric cancer and prostate cancer 33, 34. Our recent findings revealed that CtBP2 could form a transcriptional complex with p300 and the transcription factor Runx2 (Runt-related transcription factor 2), thereby regulating the expression of many genes involved in bone development and differentiation 35. Although CtBPs can interact with multiple partners, they have a conserved mechanism in which CtBPs bind other proteins through a short motif known as the PXDLS (Proline-X-Aspartate-Leucine-Serine, where X represents any amino acid) motif 32. Several studies have reported that in addition to their inhibitory roles, CtBPs have transactivation abilities to regulate gene expression 36-38. For example, CtBP2 associates with KLF8 (Kruppel-like factor 8) to activate the expression of *Tiam1* (T-cell lymphoma invasion and metastasis 1), thereby promoting cancer cell migration 36. CtBP1 is involved in the transactivation of *MDR1* (Multidrug Resistance 1) in human multidrug-resistant cancer cells 37. CtBP1 associates with LSD1 (Lysine Demethylase 1), RREB1 (RAS-responsive element-binding protein 1), PCAF (P300/CBP-associated factor) and CoREST (REST Corepressor 1) to form a complex, which can activate the expression of *NeuroD1* in gastrointestinal endocrine cells 38. Thus, CtBPs have both transrepression and transactivation roles in the regulation of gene expression, which is indicative of their complicated roles. Although CtBPs are differentially expressed in many diseases, the molecular mechanisms of their overexpression are still unclear. Some microRNAs (miRNAs) are involved in the regulation of CtBPs and their downstream events. For example, miR-137 functions as a tumor suppressor and it can target *CtBP1* directly to inhibit EMT (epithelial-mesenchymal transition) and induce apoptosis in melanoma cells 39. Moreover, miR-212 targets *CtBP1* in human endometrial epithelial cells to enhance spheroid attachment *in vitro* 40.

Recently, we found that *CtBP2* but not *CtBP1* was overexpressed in atrophic nonunion tissues 35. The amplified CtBP2 assembled a transcriptional complex with p300, and Runx2 35. This complex could medicate the expression of multiple genes, such as *COL1A1 (*Collagen 1a1*), OSC* (Osteocalcin), *IBSP* (Integrin binding sialoprotein), *ALPL* (Alkaline phosphatase), *MMP9* (Matrix metallopeptidase 9), *MMP13*, and *SPP1* (Osteopontin) 35. To explore if CtBPs function in the pathogenesis of OA, we examined the expression levels of *CtBPs* in 48 OA specimens. Our results showed that both *CtBP1* and *CtBP2* were significantly upregulated. By knocking down and overexpressing *CtBPs in vitro* and then analyzing differentially expressed genes that were dependent on *CtBPs* using a microarray analysis, we found that the expression of *NLRP3* was changed with *CtBP* overexpression or downregulation. Therefore, we will investigate how CtBPs activate *NLRP3* and explore the molecular mechanism of *CtBP* overexpression in this study.

## Materials and Methods

### Cell lines and cell culture

Human osteoarthritic chondrocyte (HC-OA, #402OA-05A) and human osteoarthritic osteoblast (HOB-OA, #406OA-05A) cell lines were obtained from Sigma-Aldrich (St. Louis, MO, USA). HC-OA cells are derived from the human articular cartilage of a donor with OA. HOB-OA cells are isolated from the bone of an OA patient. HC-OA cells were cultured in chondrocyte growth medium (PromoCell, Heidelberg, Germany, #C-27101) supplemented with 10% fetal bovine serum (FBS) (Sigma-Aldrich, #F9423), 1% penicillin-streptomycin (PS) (ThermoFisher Scientific, Waltham, MA, #10378016). HOB-OA cells were grown in HOB Basal Medium (Cell Application, Inc., San Diego, CA, USA, #416-500) supplemented with 10% FBS and 1% PS. Cells were placed in a 37°C humidified incubator containing 5% CO_2_.

### Biopsy sample collection

Cartilage tissues were obtained from 48 OA patients who had severe symptoms and needed surgical treatments to replace cartilage by an artificial endoprosthesis. At the same time, biopsies were also collected from another group of patients (n=48) who had no OA symptoms but damaged their joints in accidents. All of these patients were treated at the Department of Orthopedics of Honghui Hospital during the period 2012-2016. The biopsy samples were acquired with written informed consent from patients following a protocol reviewed and approved by the ethical board of Xi'an Jiaotong University. The basic information of these patients is included in Supplementary Table-1.

### Total RNA isolation and quantitative real-time PCR (qRT-PCR) analysis

Total RNA was isolated from tissues and cultured cells using TRIzol (Sigma-Aldrich, #T9424) following a protocol provided by the manufacturer. The purified RNA (1.0 μg) was used to synthesize cDNA using a PrimeScript™ 1st strand cDNA Synthesis Kit (TaKaRa, Dalian, China, #6110B). The resulting cDNA was diluted 10-fold and then used as a template to detect individual gene expression by qRT-PCR with a TB Green® Advantage® qPCR Premix Kit (TaKaRa, #639676). The primers used for qRT-PCR are listed in Supplementary Table-2.* β-Actin* was set as an internal control. The relative expression levels of genes were calculated using the 2*^-^*^Δ*Ct*^ method in which Δ*Ct=Ct^Gene^-Ct^Actin^.*

### Western blotting analysis

The immunoblot analysis was performed as described previously 35. Briefly, tissues and cells were lysed in 1×RIPA buffer (Sigma-Aldrich, #R0278). Equal amounts of protein in each sample were loaded into a 10% SDS-PAGE gel. After transferring to a membrane and blocking with 5% milk, the proteins were probed with the following primary antibodies: anti-CtBP1 (BD Biosciences, San Jose, CA, USA, #612042), anti-CtBP2 (BD Biosciences, #612044), anti-CD31 (ThermoFisher Scientific, #PA5-16301), anti-CD55 (ThermoFisher Scientific, #PA5-82005), anti-CD68 (ThermoFisher Scientific, #MA5-13324), anti-GAPDH (Santa Cruz Biotechnology, Dallas, Texas, USA, #sc-365062), anti-Caspase-1 (Santa Cruz Biotechnology, #sc-56036), anti-Flag (Sigma-Aldrich, #SAB4200071), anti-Myc (Abcam, Cambridge, MA, USA, #ab9106), anti-p300 (Santa Cruz Biotechnology, #sc-585), anti-c-Jun (Sigma-Aldrich, #SAB4501606), anti-c-FOS (Sigma-Aldrich, #F7799), anti-p50 (ThermoFisher Scientific, #PA1-30409), anti-p65 (ThermoFisher Scientific, #14-6731-81), anti-IRF2 (Abcam, #ab3388), anti-STAT4 (Abcam, #ab68156), anti-NLRP3 (Abcam, #ab210491), anti-Il-1β (Abcam, #ab2105), anti-DNMT1 (Abcam, #ab13537), and anti-DNMT3A (Abcam, #ab2850). After probing with secondary antibodies, protein band signals were detected using a Pierce^TM^ ECL western blotting substrate (ThermoFisher Scientific, #32106).

### Immunohistochemistry (IHC) staining and immunofluorescence (IF) assays

The IHC staining assay was performed following a standard procedure. Briefly, tissues from OA patients and healthy controls were fixed in 10% PBS-buffered formalin for 48 h. The resulting tissues were then performed a series of procedures including dehydration, infiltration and embedding in paraffin, section, and re-dehydration. The obtained slides were probed with primary antibodies including anti-CtBP1, anti-CtBP2, anti-CD31, anti-CD55, and anti-CD68, respectively, followed by incubation with secondary biotinylated antibodies for 1 h at room temperature. The mouse and rabbit specific HRP/DAB detection kit (Abcam, #ab64264) was used to determine the signals of each protein. For IF assay, the HOB-OA cells were fixed with 4% formaldehyde in PBS at room temperature for 20 min. After rinsing five times with PBS buffer, cells were incubated with the primary antibodies including anti-CtBP1, anti-p300 and anti-c-Jun, respectively, followed by incubation with Alexa Fluor 594 (red) (ThermoFisher Scientific, #A32740) and 488 (green) (ThermoFisher Scientific, #A32723) conjugated secondary antibodies at room temperature for 1 h. Cells were also stained with the fluorescent 4',6-diamidino-2-phenylindole (DAPI) to visualize the nucleus.

### Vector construction

The coding sequences of *CtBP1*, *CtBP2*, *p50*, *p65*, *STAT4*, *c-Jun*, *c-FOS*, *IRF2*, *p300*, *DNMT1*, and *DNMT3A* were cloned into the BamHI and EcoRI sites of a pCDNA3-2×Flag vector, respectively. The coding sequences of *CtBP1*, *CtBP2*, *c-Jun*, *c-FOS*, and *p300* were cloned into the BamHI and EcoRI sites of a pCDNA3-6-Myc vector, respectively. The pCDNA3-2×Flag-p300 vector was used as a template to create its mutant vector pCDNA3-2×Flag-p300^Mut^ in which the PMDLS motif was mutated to AMAAS. The promoter of *NLRP3* was cloned into the KpnI and XhoI sites of a luciferase vector pGL4.26. The pGL4.26-pNLRP3 vector was used as a template to create its mutant vector pGL4.26-pNLRP3^Mut^ in which the TGAGTCA sequences were mutated to TCCAGCA. All primers used for vector constructions were listed in Supplementary Table-3.

### Cell transfection

Cells were seeded into 6-well plates at a density of 4×10^5^ and then further grown for another 30 h to reach nearly 80% confluence. Cells were washed twice with PBS buffer and incubated in the fresh chondrocyte growth medium. A total of 1 μg plasmid DNA or 20 pM siRNAs was mixed completely with 100 μL Opti-MEM (ThermoFisher Scientific, #31985062). In addition, 5 μL Lipofectamine 2000 (ThermoFisher Scientific, #11668019) was mixed with 95 μL Opti-MEM. Then, the DNA-Lipofectamine 2000 complexes were added into each well containing cells and medium. After completely mixing, the cells were incubated at 37°C for 48 h until further experiments. For knockdown analysis of each gene, two independent siRNAs targeting different regions of genes were used. All siRNAs were ordered from Sigma-Aldrich and they included siCtBP1 (#SASI_Hs01_00243819 and # SASI_Hs02_00306193), siCtBP2 (#SASI_Hs02_00317301 and #SASI_Hs02_00317304), sip50 (#SASI_Hs02_00336720 and #SASI_Hs01_00227679), sip65 (#SASI_Hs01_00171090 and #SASI_Hs01_00171091), siSTAT4 (#SASI_Hs01_00175605 and SASI_Hs01_00175608), sic-Jun (#SASI_Hs01_00150279 and #SASI_Hs01_00150282), si-c-FOS (#SASI_Hs01_00184572 and # SASI_Hs01_00184573), siIRF2 (#SASI_Hs02_00333421 and # SASI_Hs01_00204944), sip300 (#SASI_Hs01_00052827 and #SASI_Hs01_00052819), siDNMT1 (#SASI_Hs01_00204021 and #SASI_Hs01_00204022), and siDNMT3A (#SASI_Hs01_00160193 and # SASI_Hs01_00160197).

### Microarray analysis

The microarray analysis procedure was the same as a previous protocol 35. Briefly, 2.0 μg total RNA from each sample was labeled with biotin. After cDNA was synthesized using a GeneChip 3' IVT Express Kit (ThermoFisher Scientific, #902416), the resulting products were fragmented and hybridized with a GeneChip Human Genome U133 Plus 2.0 (ThermoFisher Scientific, #900466) at 45°C for 12 h. The chip was stained with streptavidin-phycoerythrin (SAPE) for 300 seconds at 35°C, followed by scanning in a GeneArray Scanner to collect data.

### Luciferase assay

The luciferase assay was carried out following a previous protocol 41. Briefly, the combinations of pGL4.26-pNLRP3 + pRL-TK-Renilla or pGL4.26-pNLRP3^Mut^ + pRL-TK-Renilla plasmids were transfected into HC-OA-c-Jun-KD (knockdown), HAC-OA-c-FOS-KD, HC-OA-c-Jun-OE (overexpression) and HC-OA-c-FOS-OE cells, respectively. Cells were further cultured at 37°C for 48 h, followed by luciferase assays with a Nano-Glo Dual-Luciferase Reporter Assay System (Promega, Madison, WI, USA, #N1610) according to the manufacturer's protocol.

### Immunoprecipitation (IP) and Co-IP analyses

For IP analyses, the pcDNA3-2×Flag-CtBP1 and pcDNA3-2×Flag-CtBP2 plasmids were transfected into HC-OA cells. After incubation for 48 h, cells were lysed with 1×RIPA buffer supplemented with a protease inhibitor cocktail (Roche, Shanghai, China, #11697498001). Cell lysates were centrifuged at 14000 rpm for 15 min, followed by incubation with anti-Flag-agarose (Sigma-Aldrich, #A4596) at 4°C for 2 h. The Flag beads were rinsed with 1×RIPA buffer four times. The resulting Flag-CtBP-associated protein complexes were subjected to immunoblot analyses. For Co-IP analysis, the procedures were the same as a previous protocol 35. Briefly, different combinations of Flag-tagged and Myc-tagged plasmids as shown in figures were co-transfected into cells, respectively. After incubation at 37°C for another 48 h, cells were lysed and incubated with anti-Flag agarose beads and anti-c-Myc agarose beads (Sigma-Aldrich, #A7470) at 4°C for 2 h. The beads were rinsed with 1×RIPA buffer four times, and protein levels were examined using western blotting assays.

### Cell treatments

HC-OA cells at less than 80% confluence were washed twice with PBS buffer and then incubated in fresh medium for 30 min. Cells were then treated with different chemicals including 5 ng/mL or 50 ng/mL TNF-α alone, 1 mM MTOB (4-methylthio-2-oxobutyric acid) + 5 ng/mL TNF-α combination, 1 mM MTOB + 50 ng/mL TNF-α combination, 5 μΜ or 10 μΜ DNA methylation inhibitor ΑZΑ (5-aza-2′-deoxycytidine) (Sigma-Aldrich, #A3656), 5 μΜ or 10 μΜ histone deacetylase inhibitor TSΑ (trichostatin A) (Sigma-Aldrich, #T8552).

### Chromatin immunoprecipitation (ChIP) assay

ChIP assay was carried out following a previous protocol 35. Briefly, cells under 80% confluence were washed twice with PBS and then crosslinked with 1% formaldehyde in PBS for 15 min. The crosslinked cells were subjected to sonication to break the DNA fragments to an average size of 300-500 bp. After centrifugation at 14000 rpm for 15 min, half of the supernatant was used as input, and the other half of supernatant was subjected to ChIP assay with a Millipore ChIP Assay Kit (Millipore, Burlington, MA, USA, #17295) according to the manufacturer's instructions. The antibodies used in the ChIP assay included anti-CtBP1, anti-CtBP2, anti-p300, anti-c-FOS and anti-c-Jun. All of these antibodies were the same as the ones used in the immunoblot analysis. The purified output DNA was subjected to qRT-PCR analyses using primers included in Supplementary Table-4. The relative enrichment was determined using the 2*^-^*^Δ*Ct*^ method in which Δ*Ct=Ct^Output^-Ct^Input^.*

### Enzyme-linked immunosorbent assay (ELISA) assay

The ELISA assays in the supernatant of cell culture were performed to measure the secreted cytokines including IL-1β, IL-6, TNF-α, and IL-4 following a previous protocol 42. Briefly, the CtBP1-OE and CtBP2-OE cells were seeded into 6-well plates and cultured at 37°C for 48 h. The medium was collected and centrifuged at 1500 rpm for 10 min and the supernatant was applied to ELISA assays using different kits including IL-1β (Αbcam, #ab214025), IL-6 (Abcam, #ab46027), TNF-α (Αbcam, #ab46087), and IL-4 (Abcam, # ab46063) following the protocols provided by the manufacturer.

### Quantitative methylation-specific PCR (qMSP) assay

The qMSP assay was carried out as described previously 43. Genomic DNA was isolated from tissues or cells using a Monarch Genomic DNA Purification Kit (New England Biotechnology, Beijing, China, #T3010L) following the manufacturer's protocol. A total of 2.0 μg genomic DNA from each sample was treated with an EZ DNA Methylation-GOLD Kit (Zymo Research, Tustin, CA, USA, #D5006) in which sodium bisulfite converted unmethylated cytosines but not methylated cytosines to uracil. The modified DNA was subjected to qMSP analysis using a TaqMan^®^ Universal Master Mix II non-UNG Kit (ThermoFisher Scientific, #4440038) with primers included in Supplementary Table-5.

### Statistical analysis

All experiments in this study were independently repeated in triplicate. The data were analyzed using two-tailed Student's *t*-tests with the SPSS software version 22 and presented by mean ± SD. The significance levels were set at *P* < 0.05 (*), *P* < 0.01 (**) and *P* < 0.001 (***).

## Results

### Both *CtBP1* and *CtBP2* were involved in the pathogenesis of OA

**Figure 1:**
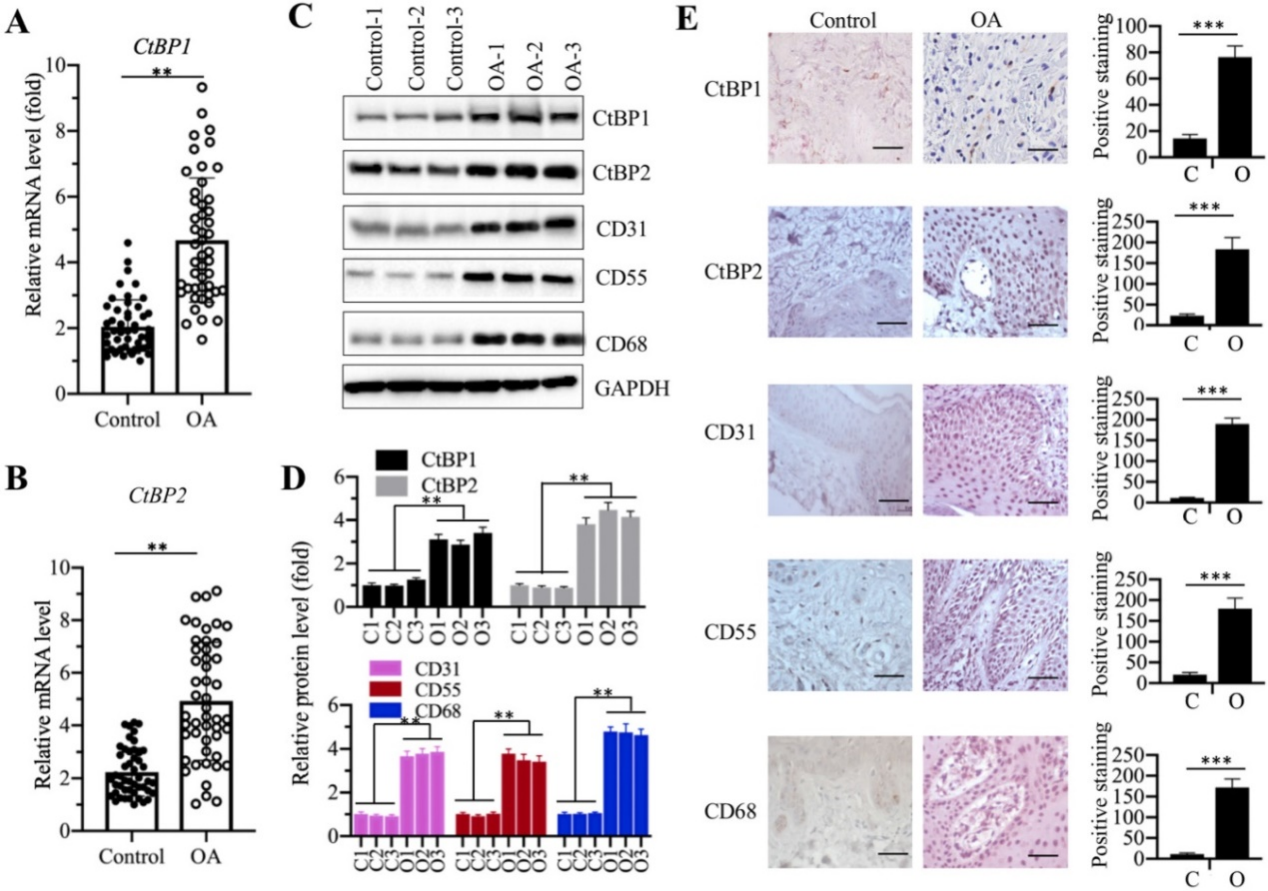
** CtBP mRNA and protein levels were significantly increased in OA biopsies**. **(A** and **B)** The relative mRNA levels of CtBP1 and CtBP2. Total RNA from biopsies of 48 OA patients and 48 controls was used to determine the mRNA levels of CtBP1 **(A)** and CtBP2 **(B)** by qRT-PCR analyses. The expression of CtBP1 and CtBP2 in a healthy control was defined as one-fold. **P<0.01.** (C** and** D)** The protein levels of CtBPs and OA markers in three-paired biopsies. Total cell extracts of three-paired biopsies from OA patients and controls were subjected to western blotting to examine the protein levels of CtBP1, CtBP2, CD31, CD55 and CD68 **(C)**. GAPDH was used as a loading control. **(D)** Statistical data of the relative protein levels of CtBPs and OA markers in (C). **P<0.01. **(E)** IHC staining results of CtBPs and OA markers. The same biopsies as (C) were used to IHC staining and representative CtBP1, CtBP2, CD31, CD55 and CD68 images were shown. Bars=100 μm. The positive cell numbers were counted and shown in the right panels. C: Control; O: OA. ***P<0.001.

Our recent publication reported that *CtBP2* was overexpressed in atrophic nonunion tissues and it repressed the expression of bone development and differentiation genes 35. To determine if CtBPs are also involved in the pathogenesis of OA, we collected 48 paired cartilage tissues from OA patients and from patients who had no OA symptoms but underwent joint surgeries because of accidents (set as controls) and measured the mRNA levels of *CtBP1* and *CtBP2* in these biopsies. The qRT-PCR results showed that both *CtBP1* and *CtBP2* mRNA levels were significantly upregulated nearly 5-fold in OA tissues compared to controls (Figures 1A and 1B). To further determine if their protein levels were also elevated, we randomly selected three paired biopsies and then examined the protein levels of CtBP1, CtBP2 and three well-known OA markers including CD31 (Cluster of differentiation), CD55 and CD68 44. Consistent with their mRNA levels, the western blotting results indicated that both CtBP1 and CtBP2 protein levels were increased ~3.5-4.2-fold (Figures 1C and 1D). The protein levels of three OA markers were also significantly increased ~3.5-4.5-fold in these three OA biopsies (Figures 1C and 1D). Meanwhile, we also performed IHC staining to determine the protein levels of CtBPs and OA markers in both controls and OA specimens. Consistent with the immunoblot results, the IHC staining results also indicated that CtBPs and OA markers were significantly induced in OA specimens. Based on these results, we next sought to determine if overexpression of *CtBPs* could cause the induction of OA markers *in vitro*. Accordingly, we transfected pCDNA3-2×Flag and pCDNA3-2×Flag-CtBP1/2 into HC-OA cells to generate the HC-OA-empty vector (EV) and CtBP overexpression cell lines (CtBP1-OE and CtBP2-OE). After determining *CtBP* mRNA levels in these cells and HOB-OA cells (Supplementary Figure 1A), we examined the protein levels of three OA markers. As shown in Supplementary Figures 1B and 1C, we found that overexpression of *CtBPs* could induce the protein levels of CD31, CD55 and CD68 up to ~3.0-fold. Given that OA markers are routinely activated by inflammation stimuli, we next sought to determine if *CtBP* overexpression could increase the secretion of proinflammatory cytokines in cell culture. Thus, we measured the concentrations of three proinflammatory cytokines including IL-1β, IL-6 and TNF-α and one anti-inflammatory cytokine IL-4 in the supernatant of the medium after culturing CtBP-OE cells. The ELISA results indicated that the secreted IL-1β, IL-6, and TNF-α were significantly increased (Supplementary Figures 1D-1F), while the concentration of IL-4 was not obviously changed in the supernatant of CtBP-OE cells (Supplementary Figure 1G). These results suggested that overexpression of *CtBPs* might increase proinflammatory cytokine levels and thus activate OA markers.

### The expression of *NLRP3* was dependent on *CtBP* levels

**Figure 2:**
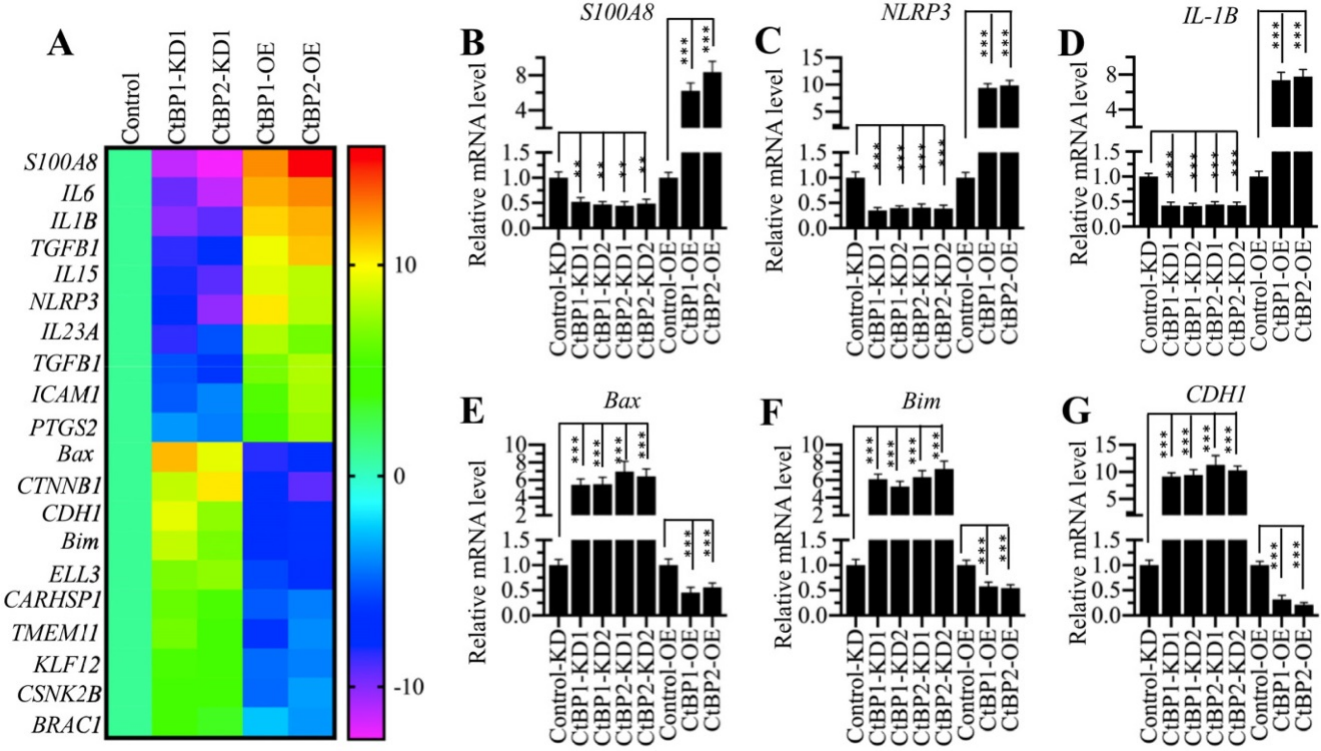
** The identification of differentially expressed genes dependent on *CtBPs*. (A)** Heatmaps of genes coregulated by *CtBP1* and *CtBP2*. Total RNA from HC-OA (control), CtBP1-KD1, CtBP2-KD1, CtBP1-OE and CtBP2-OE cells were subjected to a microarray analysis. Genes coregulated by *CtBP1* and *CtBP2* were shown. **(B-G)** Examination of the expression levels of genes identified in the microarray analysis. Six genes including *S100A8* **(B)**, *NLRP3* **(C)**, *IL1B* **(D)**, *Bax*, **(E)** *Bim* **(F)**, and *CDH1* **(G)** were selected to verify their expression in HC-OA-siControl (Control-KD), CtBP1-KD1, CtBP1-KD2, CtBP2-KD1, CtBP2-KD2, HC-OA-pCDNA3-2×Flag (Control-OE), CtBP1-OE and CtBP2-OE cells. The expression of these genes in controls was defined as one-fold. **P<0.01 and ***P<0.001.

CtBPs function as transcriptional regulators to control gene expression in different biological processes 32. To determine the genes regulated by CtBPs in the pathogenesis of OA, we conducted a microarray analysis in CtBP-KD and CtBP-OE cells. Accordingly, we created two independent cell lines of CtBP1-KD and CtBP2-KD, respectively, one CtBP1-OE and one CtBP2-OE cell lines. After verifying the successful knockdown and overexpression of *CtBPs* in these cells (Supplementary Figure 2), we subjected total RNA from HC-OA, CtBP1-KD1, CtBP2-KD1, CtBP1-OE and CtBP2-OE cells to a microarray analysis. In total, we identified 58 genes controlled by *CtBP1* (Supplementary Table-6) and 73 genes regulated by *CtBP2* (Supplementary Table-7) (Supplementary Figure 3), respectively. Comparing these two gene lists, we surprisingly found that a total of 29 overlapping genes (Supplementary Table-6 and -7). As shown in Figure 2A, we generated a heatmap of 20 genes that were both dependent on *CtBP1* and *CtBP2*. Of these genes, we found several important genes including *NLRP3*, which is a critical member of the inflammasome, *IL-1B* and *IL-6*, two critical proinflammatory cytokines. These results further supported our above hypothesis in which CtBPs were involved in the pathogenesis of OA by increasing proinflammatory cytokines and thus activating OA markers. To test the accuracy of our microarray results, we randomly selected 6 genes including* NLRP3*, *IL-1B*, *S100A8*, *Bax*, *Bim* and *CDH1* and examined their expression in CtBP-KD and CtBP-OE cells. Consistent with the microarray results, we found that the expression of *NLRP3*, *IL-1B* and *S100A8* was increased with *CtBP* overexpression but decreased with *CtBP* knockdown (Figures 2B-2D). Conversely, the expression of *Bax*, *Bim* and *CDH1* was decreased with *CtBP* overexpression but increased with *CtBP* knockdown (Figures 2E-2G).

### Overexpression of *CtBPs* caused the activation of NLRP3 downstream signaling

**Figure 3:**
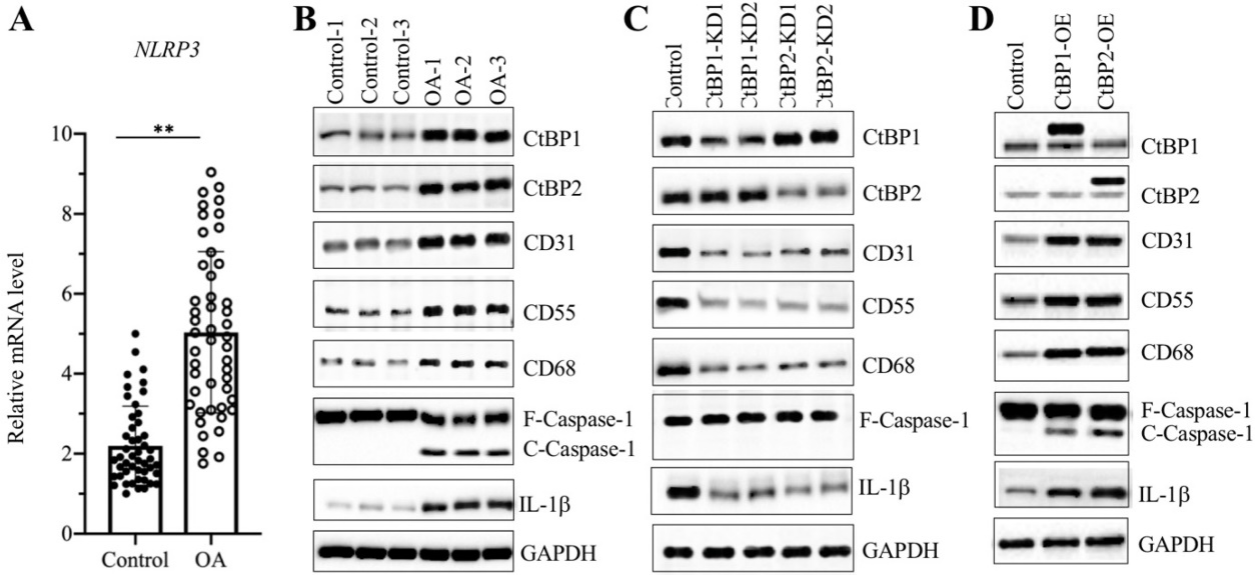
** NLRP3 and its downstream signaling were dependent on CtBPs. (A)** The relative mRNA levels of *NLRP3*. The mRNA level of *NLRP3* was measured in 48-paired biopsies from OA patients and controls by qRT-PCR analyses. The expression of *NLRP3* in a healthy control was defined as one-fold. **P<0.01. **(B)** NLRP3 and its downstream signaling were activated in OA biopsies. Total cell extracts of three-paired biopsies from OA patients and controls were subjected to western blotting to examine the protein levels of CtBP1, CtBP2, CD31, CD55, CD68, NLRP3, Caspase-1 and IL-1β. GAPDH was used as a loading control. **(C)** The downregulation of *CtBPs* inactivated NLRP3 and its downstream signaling *in vitro*. Total proteins isolated from Control-KD, CtBP1-KD1, CtBP1-KD2, CtBP2-KD1 and CtBP2-KD2 cells were used for western blotting analyses to measure the protein levels of CtBP1, CtBP2, CD31, CD55, CD68, NLRP3, Caspase-1 and IL-1β. GAPDH was used as a loading control. **(D)** Overexpression of *CtBPs* activated NLRP3 and its downstream signaling *in vitro*. Total proteins isolated from Control-OE, CtBP1-OE and CtBP2-OE cells were used for western blotting analyses to measure the protein levels of CtBP1, CtBP2, CD31, CD55, CD68, NLRP3, Caspase-1 and IL-1β. GAPDH was used as a loading control.

Since we observed overexpression of *CtBPs* in OA biopsies and found that the expression of *NLRP3* was dependent on *CtBP* levels, we next sought to examine the mRNA level of *NLRP3* in OA biopsies. The qRT-PCR results showed that the average *NLRP3* mRNA level was upregulated ~5-fold in OA tissues compared to controls (Figure 3A). It is well known that the NLRP3-associated inflammasome can activate Caspase-1, which subsequently leads to the processing of IL-1β 45. Thus, the significant increase in *NLRP3* in OA biopsies would also activate its downstream signaling. For this purpose, we examined NLRP3, Caspase-1 and IL-1β levels in three-paired tissues from OA patients and controls. As expected, the western blotting results showed that all these three proteins were activated in OA tissues but not in controls (Figure 3B and Supplementary Figure 4A). Meanwhile, we also detected CtBPs and OA makers in these tissues. The immunoblot results consistently showed that both CtBPs and OA makers were induced in OA tissues compared to controls (Figure 3B and Supplementary Figure 4A). To further evaluate whether the activation of NLRP3 and Caspase-1 was dependent on CtBPs, we also determined NLRP3, Caspase-1 and IL-1β levels in CtBP-KD and CtBP-OE cells. The results indicated that both NLRP3, cleaved Caspase-1 and IL-1β protein levels were increased in CtBP-OE cells but were decreased in CtBP-KD cells (Figures 3C, 3D and Supplementary Figures 4B and 4C). Interestingly, the similar expression patterns in OA markers were also observed in these cells (Figures 3C, 3D and Supplementary Figures 4B and 4C).

### Transcription factor AP1 specifically regulated the expression of *NLRP3 in vitro*

**Figure 4:**
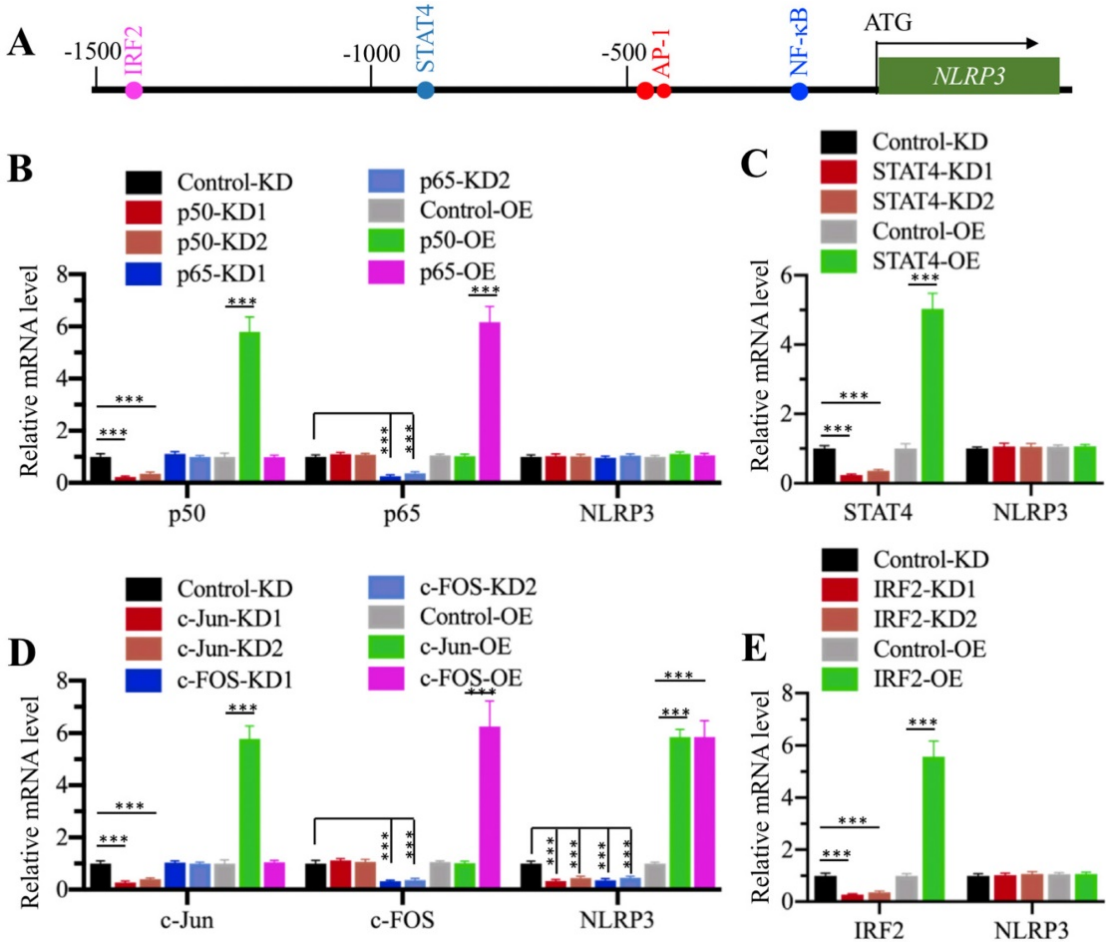
** AP1 specifically regulated the expression of *NLRP3*. (A)** Transcription factor binding sites in the promoter of *NLRP3*. A 1500-bp region in the *NLRP3* promoter was used to analyze transcription factor binding sites, and one NF-κB, two AP1, one STAT4 and one IRF4 binding sites were found. Their binding positions were indicated. **(B)** Effects of knockdown and overexpression of *NF-κB* subunits on *NLRP3* expression. The Control-KD, p50-KD1, p50-KD2, p65-KD1, p65-KD2, Control-OE, p50-OE and p65-OE cells were used to examine the mRNA levels of *p50*, *p65* and *NLRP3*. The expression of these genes in controls was defined as one-fold. ***P<0.001. **(C)** Effects of knockdown and overexpression of *STAT4* on *NLRP3* expression. The Control-KD, STAT4-KD1, STAT4-KD2, Control-OE and STAT4-OE cells were used to examine the mRNA levels of *STAT4* and *NLRP3*. The expression of these genes in controls was defined as one-fold. ***P<0.001. **(D)** Effects of knockdown and overexpression of AP-1 subunits on *NLRP3* expression. The Control-KD, c-Jun-KD1, c-Jun-KD2, c-FOS-KD1, c-FOS-KD2, Control-OE, c-Jun-OE and c-FOS-OE cells were used to examine the mRNA levels of *c-Jun*, *c-FOS* and *NLRP3*. The expression of these genes in controls was defined as one-fold. ***P<0.001. **(E)** Effects of knockdown and overexpression of *IRF2* on *NLRP3* expression. The Control-KD, IRF2-KD1, IRF2-KD2, Control-OE and IRF2-OE cells were used to examine the mRNA levels of *IRF2* and *NLRP3*. The expression of these genes in controls was defined as one-fold. ***P<0.001.

CtBPs only function as transcriptional coregulators, and they need to associate with other proteins to form a transcriptional complex 32. To determine the transcription factor that regulates *NLRP3* expression, we primarily analyzed the transcription factor binding sites in a 1500-bp region of the *NLRP3* promoter. We found one NF-κB site [GGGGTGCCTC, -179-(-)188], two AP1 sites [TGAGTCA, -470-(-476) and -477-(-)483], one STAT4 site [AATTCC, -838-(-)844], and one IRF2 (Interferon regulatory factor 2) site [AAGTGA, -1422-(-)1428] (Figure 4A). To determine if any of these transcription factors could directly regulate the expression of *NLRP3*, we individually knocked down and overexpressed these transcription factors in HC-OA cells and then examined *NLRP3* mRNA levels. After knockdown or overexpression of two NF-κB subunits including *p65* and *p50*, we did not observe a significant change in *NLRP3* mRNA level (Figure 4B and Supplementary Figures 5A and 5B). Similarly, we also did not identify a significant change in *NLRP3* mRNA level in STAT4-KD and STAT4-OE cells (Figure 4C and Supplementary Figures 5C and 5D). However, *NLRP3* mRNA level was significantly decreased when AP1 subunits (*c-Jun* and *c-FOS*) were knocked down and dramatically increased when AP1 subunits were overexpressed (Figure 4D and Supplementary Figures 5E and 5F). In addition, we also found that knockdown or overexpression of *IRF2* did not affect *NLRP3* mRNA level (Figure 4E and Supplementary Figures 5G and 5H). These results suggest that AP1 subunits could specifically regulate the expression of *NLRP3 in vitro*. To further determine if AP1 subunits bound to the promoter of *NLRP3* through the consensus sequence TGAGTCA, we constructed a mutant in which both two AP1 sites located in -470-(-476) and -477-(-)483 were changed to TCCAGCA. We co-transfected pGL4.26-pNLRP3^WT^ + Renilla or pGL4.26-pNLRP3^Mut^ + Renilla into c-Jun-KD, c-FOS-KD, c-Jun-OE, and c-FOS-OE cells, respectively. After determining the expression of *c-Jun* and *c-FOS* in these cells (Supplementary Figures 6A and 6B), we performed a luciferase assay to compare the changes in luciferase activities. In cells expressing pGL4.26-pNLRP3^WT^ + Renilla, knockdown of either *c-Jun* or *c-FOS* could significantly decrease the luciferase activities, while overexpression of either *c-Jun* or *c-FOS* markedly induced the luciferase activities (Supplementary Figures 6C and 6D). In contrast, we found that knockdown or overexpression of *c-Jun* or *c-FOS* could not significantly change the luciferase activities in cells expressing pGL4.26-pNLRP3^Mut^ + Renilla (Supplementary Figures 6C and 6D). These results clearly suggested that AP1 subunits bound to the promoter of *NLRP3* through the consensus sequence TGAGTCA.

### CtBPs, p300 and AP1 subunits assembled a transcriptional complex

**Figure 5:**
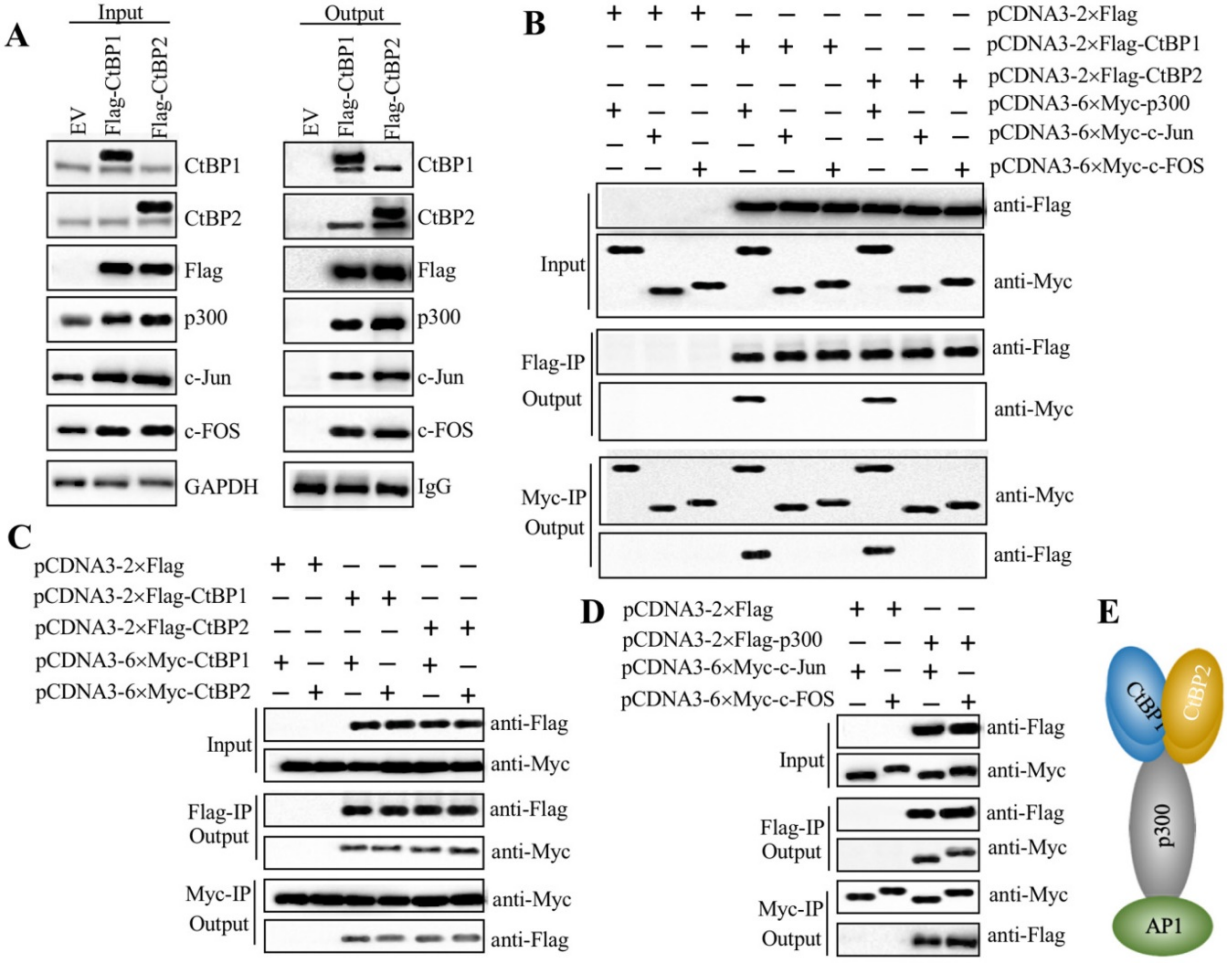
** AP1 recruited p300 and CtBPs to form a transcriptional complex *in vitro*. (A)** Both CtBP1 and CtBP2 associated with p300 and AP1. HC-OA cells were transfected with pcDNA3-2×Flag empty vector (EV), pcDNA3-2×Flag-CtBP1, or pcDNA3-2×Flag-CtBP2. The resulting cells were lysed, and 1/10 total cell extracts were taken out as input, and the other 9/10 cell extracts were subjected to IP analysis with an anti-Flag agarose. The input (left panel) and output (right panel) proteins were subjected to western blotting to examine protein levels using anti-Flag, anti-CtBP1, anti-CtBP2, anti-p300, anti-c-Jun and anti-c-FOS antibodies, respectively. GAPDH and IgG were used as the loading control of input and output proteins, respectively. **(B)** CtBPs directly interacted with p300 instead of AP1 subunits *in vitro*. Different combinations of plasmids including pCDNA3-2×Flag + pCDNA3-6×Myc-p300, pCDNA3-2×Flag + pCDNA3-6×Myc-c-Jun, pCDNA3-2×Flag + pCDNA3-6×Myc-c-FOS, pCDNA3-2×Flag-CtBP1 + pCDNA3-6×Myc-p300, pCDNA3-2×Flag-CtBP1 + pCDNA3-6×Myc-c-Jun, pCDNA3-2×Flag-CtBP1 + pCDNA3-6×Myc-c-FOS, pCDNA3-2×Flag-CtBP2 + pCDNA3-6×Myc-p300, pCDNA3-2×Flag-CtBP2 + pCDNA3-6×Myc-c-Jun, and pCDNA3-2×Flag-CtBP2 + pCDNA3-6×Myc-c-FOS were cotransfected into HC-OA cells. After incubating for another 48 h, cells were lysed and 1/10 total cell extracts were taken out as input, and the other 9/10 cell extracts were subjected to IP analysis with an anti-Flag agarose and anti-Myc agarose, respectively. The input and output proteins were probed with anti-Flag and anti-Myc antibodies, respectively.** (C)** CtBPs formed a heterotetramer *in vitro*. Different combinations of plasmids including pCDNA3-2×Flag + pCDNA3-6×Myc-CtBP1, pCDNA3-2×Flag + pCDNA3-6×Myc-CtBP2, pCDNA3-2×Flag-CtBP1 + pCDNA3-6×Myc-CtBP1, pCDNA3-2×Flag-CtBP1 + pCDNA3-6×Myc-CtBP2, pCDNA3-2×Flag-CtBP2 + pCDNA3-6×Myc-CtBP1, and pCDNA3-2×Flag-CtBP2 + pCDNA3-6×Myc-CtBP2 were cotransfected into HC-OA cells. After incubating for another 48 h, cells were lysed and 1/10 total cell extracts were taken out as input, and the other 9/10 cell extracts were subjected to IP analysis with an anti-Flag agarose and anti-Myc agarose, respectively. The input and output proteins were probed with anti-Flag and anti-Myc antibodies, respectively. **(D)** p300 directly interacted with AP1 subunits *in vitro*. Different combinations of plasmids including pCDNA3-2×Flag + pCDN3-6×Myc-c-Jun, pCDNA3-2×Flag + pCDNA3-6×Myc-c-FOS, pCDNA3-2×Flag-p300 + pCDNA3-6×Myc-c-Jun, and pCDNA3-2×Flag-p300 + pCDNA3-6×Myc-c-FOS were cotransfected into HC-OA cells. After incubating for another 48 h, cells were lysed and 1/10 total cell extracts were taken out as input, and the other 9/10 cell extracts were subjected to IP analysis with an anti-Flag agarose and anti-Myc agarose, respectively. The input and output proteins were probed with anti-Flag and anti-Myc antibodies, respectively. **(E)** A schematic model of the CPAC transcriptional machinery. AP1 subunits directly interacted with p300, which recruited the CtBP heterotetramer to assemble a complex.

Since both CtBPs and AP1 could activate the expression of *NLRP3*, we next evaluated whether they could directly form a transcriptional complex. Based on the notion that CtBPs interact with other proteins through a conserved PXDLS motif 32, we primarily analyzed the amino acid sequences of c-Jun and c-FOS to search if they contained this conserved motif or similar motifs, such as PXDLX, XXDLS and PXDXS. Unfortunately, we were unable to identify any of these motifs (Supplementary Figure 7). Based on the notion that transcription factors commonly interact with HATs such as p300 and CBP to activate gene expression, and CtBPs have been shown to directly interact with p300 through the PXDLS motif 28, 32, we proposed that p300 could function as a linker to directly interact with both CtBPs and AP1, thus forming a complex. To verify this hypothesis, we performed IP assays using CtBP1-OE and CtBP2-OE cells in the HC-OA background. After purification, the Flag-CtBP1- and Flag-CtBP2-associated protein complexes were subjected to western blotting analyses to detect if they could pull down p300 and AP1 subunits. As expected, our results indicated that both CtBP1 and CtBP2 could pull down p300, c-Jun and c-FOS (Figure 5A), which suggested that they could assemble a CtBP-p300-AP1 complex (CPAC). Moreover, CtBP1 and CtBP2 could also pull down themselves and each other (Figure 5A), which suggested that they might form a heterotetramer. To determine the direct interactions between CtBPs and p300, CtBPs and AP1 subunits, p300 and AP1 subunits, as well as CtBP1 and CtBP2, we constructed the Flag- and Myc-tagged vectors of these proteins and then performed Co-IP assays to examine how the CPAC transcriptional machinery was assembled. The western blotting results showed that CtBPs could directly interact with p300 instead of AP1 subunits (Figure 5B). Moreover, CtBP1 and CtBP2 could also interact with themselves and each other *in vitro* (Figure 5C). The AP1 subunits could directly interact with p300 (Figure 5D). These results suggested that p300 functioned as a linker to interact with CtBPs and AP1 subunits, thereby assembling the CPAC transcriptional machinery (Figure 5E). To further verify the formation of CPAC, we overexpressed *CtBPs* in p300-KD cells and then performed IP analyses to determine if the output of AP1 subunits was changed. As shown in Supplementary Figure 8A, equal amounts of Flag-CtBPs pulled down much less p300 and AP1 subunits in the p300-KD background cells compared to the HC-OA background cells. To determine if CtBPs interacted with p300 through the PXDLS motif, we created a p300 mutant in which the PMDLS motif was mutated to AMAAS and then examined the direct interactions between CtBPs and p300^Mut^, and AP1 subunits and p300^Mut^. The immunoblot results indicated that CtBPs could not interact with p300^Mut^, while the interaction between AP1 subunits and p300^Mut^ was not changed in comparison to the wild type p300 (Supplementary Figure 8B). These results suggested that p300 interacted with CtBPs and AP1 subunits through different domains. In addition, we also carried out IF analyses to determine if CtBPs, p300 and AP1 subunits were colocalized in the nucleus. Accordingly, we examined the colocalizations of CtBP1 and p300, p300 and c-Jun, and CtBP1 and c-Jun. Our results indicated that these three paired proteins were colocalized in the nucleus (Supplementary Figure 8C).

### Knockdown of *CtBPs*, *p300* or *AP1* subunits decreased their bindings to the promoter of *NLRP3*

Our above results indicated that CtBPs and AP1 were required for *NLRP3* transactivation, we next also aimed to evaluate whether p300 had a similar effect. Consequently, we knocked down and overexpressed *p300* in HC-OA cells and examined the mRNA levels of *NLRP3*. The qRT-PCR results indicated that knockdown of *p300* also caused a decrease in *NLRP3* mRNA level, while overexpression of *p300* resulted in its induction (Supplementary Figure 9A). We next sought to determine if the CPAC was able to directly bind to the *NLRP3* promoter. For this purpose, we performed ChIP assays in CtBP-OE and CtBP-KD cells using anti-CtBP1, anti-CtBP2, anti-p300, anti-c-Jun and anti-c-FOS antibodies. Our results showed that the enrichment of the individual members of CPAC in the *NLRP3* promoter was significantly increased in CtBP1-OE cells but dramatically decreased in CtBP-KD cells (Supplementary Figures 9B and 9C).In addition, we also performed ChIP assays in p300-KD, p300-OE, c-Jun-KD, c-Jun-OE, c-FOS-KD and c-FOS-OE cells using anti-CtBP1, anti-CtBP2, anti-p300, anti-c-Jun and anti-c-FOS antibodies. Similarly, we also observed that the enrichment of CPAC members in the *NLRP3* promoter was significantly increased in the overexpressed cells but dramatically decreased in the knockdown cells (Supplementary Figures 10A and 10B). These results clearly indicated that the CPAC transcriptional machinery specifically bound to the promoter of *NLRP3* and activated its expression.

### Inhibition of CtBPs with the specific inhibitor MTOB impaired *NLRP3* expression

**Figure 6:**
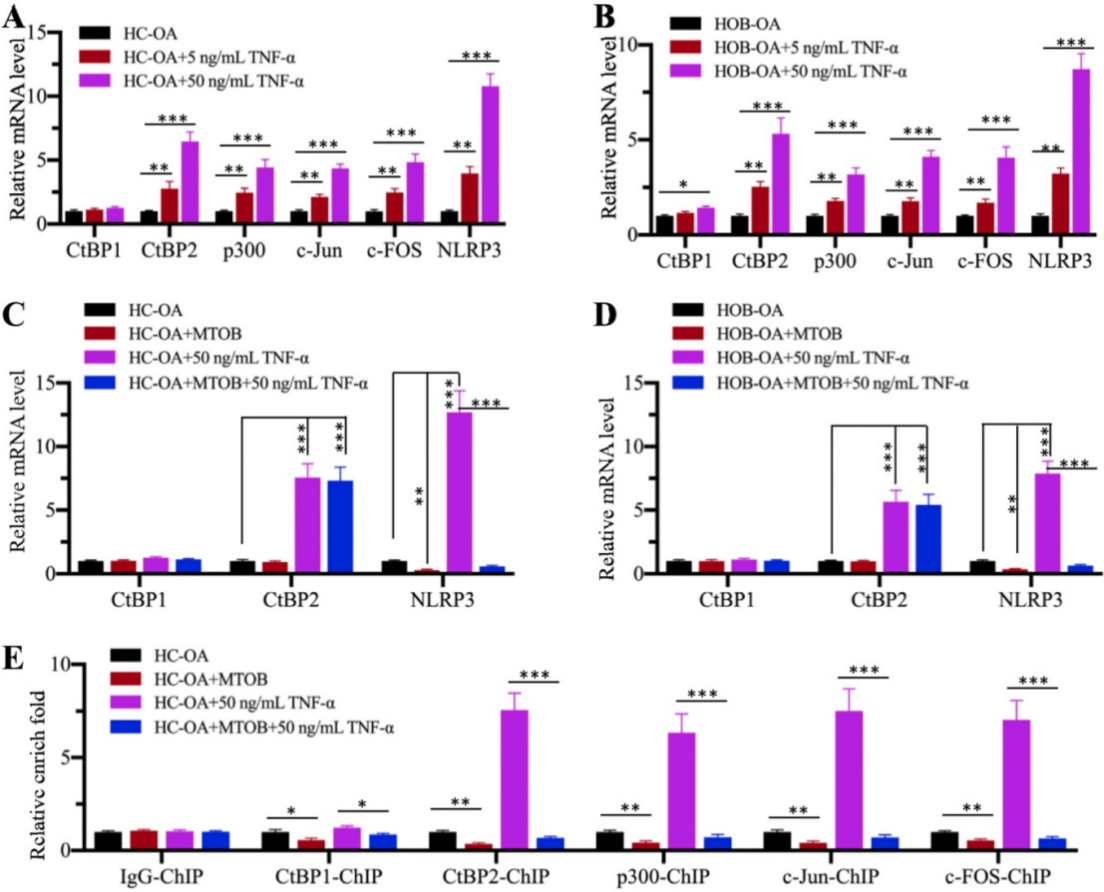
** TNF-α activated the CPAC *in vitro* and the blockage of CtBPs inhibited *NLRP3* expression. (A** and **B)** Effects of TNF-α on the expression of CPAC members and *NLRP3*. HC-OA **(A)** and HOB-OA **(B)** cells were treated with different concentrations of TNF-α including 0, 5 and 50 ng/mL for 6 h, followed by RNA isolation and qRT-PCR analyses to examine the mRNA levels of *CtBP1*, *CtBP2*, *p300*, *c-Jun*, *c-FOS* and *NLRP3*. **P<0.01 and ***P<0.001.** (C** and **D)** Effects of CtBP inhibition on the expression of *NLRP3*. HC-OA **(C)** and HOB-OA **(D)** cells were treated with 1 mM MTOB or 50 ng/mL TNF-α alone or together for 6 h. The resulting cells were subjected to RNA isolation and qRT-PCR analyses to examine the mRNA levels of *CtBP1*, *CtBP2* and *NLRP3*. **P<0.01 and ***P<0.001.** (E)** Effects of CtBP inhibition on the enrichment of CPAC members in the promoter of *NLRP3*. Cells used in (C) were subjected to ChIP assays with IgG, anti-CtBP1, anti-CtBP2, anti-p300, anti-c-Jun or anti-c-FOS antibody, followed by qRT-PCR analyses to determine the enrichment of these proteins in the *NLRP3* promoter. The enrichment of CPAC members in untreated HC-OA cells was defined as one-fold. *P<0.05, **P<0.01 and ***P<0.001.

Since CtBPs functioned as activators to regulate *NLRP3* expression, we speculated that the blockage of CtBP function should inhibit *NLRP3* expression. For this purpose, we treated HC-OA and HOB-OA cells with the proinflammatory cytokine TNF-α to mimic inflammation *in vitro*. Our results showed that *CtBP2*, *p300*, *c-Jun*, *c-FOS* and *NLRP3* mRNA levels were upregulated with TNF-α treatment in a dose-dependent manner (Figures 6A and 6B). However, we only observed a slight induction of *CtBP1* mRNA level in HOB-OA cells but not in HC-OA cells with TNF-α treatment (Figures 6A and 6B). Next, we treated HC-OA and HOB-OA cells with the CtBP inhibitor MTOB prior to TNF-α stimulation and measured the expression of *CtBPs* and *NLRP3*. As shown in Figures 6C and 6D, inhibition of CtBP functions did not change the mRNA levels of *CtBP1* and *CtBP2* but significantly decreased the expression of *NLRP3*. To further evaluate whether MTOB treatment could decrease the binding of CPAC members to the promoter of *NLRP3*, we carried out ChIP assays in cells treated with both MTOB and TNF-α using anti-CtBP1, anti-CtBP2, anti-p300, anti-c-Jun and anti-c-FOS antibodies. Our results indicated that the enrichment of CPAC members in the *NLRP3* promoter was significantly decreased in cells treated with MTOB+TNF-α compared to cells only treated with TNF-α (Figure 6E). These results suggested that the blockage of CtBPs with the specific inhibitor MTOB impaired the binding of CPAC to the promoter of *NLRP3* and thus inhibited its expression.

### Decreased DNA methylation levels in the promoters of *CtBPs* caused their overexpression in OA biopsies

**Figure 7:**
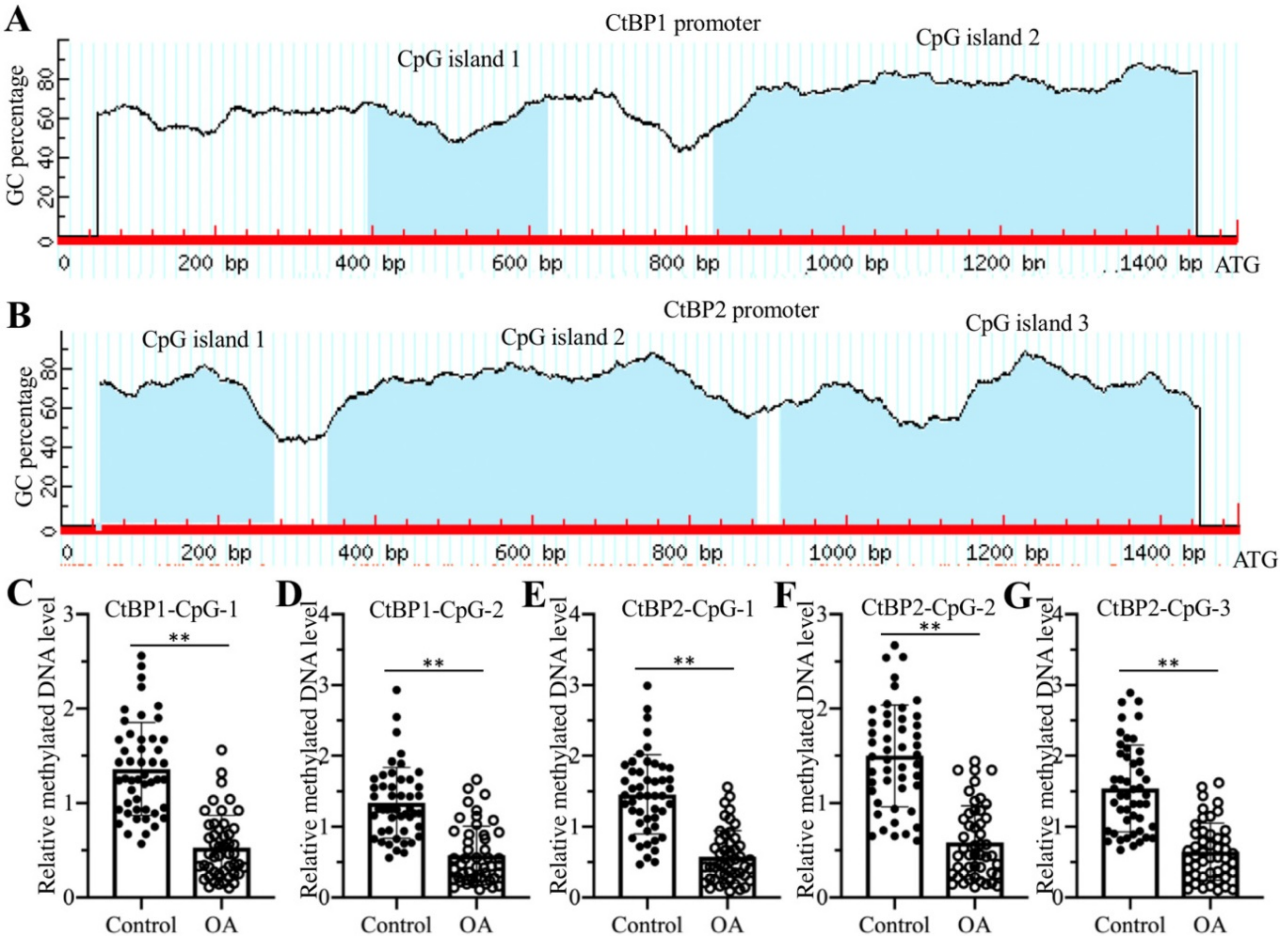
** DNA methylation levels in the promoters of *CtBP*s were decreased in OA biopsies. (A** and **B)** The promoters of *CtBPs* were abundant in CpG islands. A 1500-bp region in the promoter of either *CtBP1* or *CtBP2* was used to analyze GC contents, and two CpG islands in the promoter of *CtBP1* **(A)** and three CpG islands in the promoter of *CtBP2* **(B)** were identified.** (C**-**G) The** DNA methylation levels of *CtBP* promoters were decreased in OA biopsies. Genomic DNA of 48-paired biopsies from OA patients and controls were treated with sodium bisulfite, followed by qMSP analyses to measure methylated DNA levels in the CpG islands of *CtBP1* **(C** and **D)** and *CtBP2* **(E-F)** promoters. ** P < 0.01.

The overexpression of *CtBP* mRNAs in OA biopsies suggested that they were regulated at the transcriptional level. To reveal the underlying mechanism of *CtBP* overexpression, we analyzed their promoters (a 1500-bp region upstream of the ATG) in a database (http://www.urogene.org) and found that the promoters of *CtBP1* and *CtBP2* contained two and three CpG islands, respectively (Figures 7A and 7B). These results suggested that DNA methylation might be involved in the regulation of *CtBP* overexpression. Following this, we treated genomic DNA from 48 OA patients and controls with sodium bisulfite and then examined DNA methylation levels using primers specifically located in each CpG island. The qMSP results showed that DNA methylation levels in all CpG islands were significantly decreased in DNA from OA patients compared to controls (Figures 7C-7G). Given that DNA methylation is mainly mediated by DNMT1 (DNA methyltransferase 1) and DNMT3, we also examined both *DNMT1* and *DNMT3A* mRNA levels in biopsies of OA patients and controls. Our results indicated that both *DNMT1* and *DNMT3A* mRNA levels were slightly downregulated in OA patients compared to controls (Supplementary Figures 11A and 11B). To determine if knockdown and overexpression of *DNMTs* could change the expression of *CtBPs* and *NLRP3*, we generated DNMT1-KD, DNMT1-OE, DNMT3A-KD and DNMT3A-OE cell lines (Supplementary Figure 11C), followed by measuring mRNA and protein levels of *CtBPs* and *NLRP3*. Our results indicated that the mRNA and protein levels of *CtBPs* and *NLRP3* were increased in DNMT1-KD and DNMT3A-KD cells but decreased in DNMT1-OE and DNMT3A-OE cells (Supplementary Figures 11D-11G). In addition, we also treated HC-OA cells with the DNA methylation inhibitor AZA and then measured *CtBP*s and *NLRP3* mRNA levels. Our results showed that both *CtBPs* and *NLRP3* mRNA levels were significantly increased with AZA treatment in a dose-dependent manner (Supplementary Figures 12A and 12B). In contrast, treatments with the histone deacetylase inhibitor TSA did not affect the expression of *CtBPs* and *NLRP3* (Supplementary Figures 12A and 12B). We also observed the similar effects of AZA and TSA treatments on the protein levels of CtBPs and NLRP3 (Supplementary Figures 12C and 12D). In addition, we also performed ChIP assays to determine the enrichment of CPAC members in the promoter of *NLRP3* in cells treated with AZA and in cells overexpressing or knocking down *DNMTs*. The ChIP assay results indicated that AZA treatment and knockdown of *DNMT1* or *DNMT3A* increased the enrichment of CPAC members in the promoter of *NLRP3* (Supplementary Figure 13), while overexpression of *DNMT1* or *DNMT3A* resulted in a decrease in the enrichment of CPAC members in the promoter of *NLRP3* (Supplementary Figure 13). These results clearly suggested that the decreased DNA methylation levels were responsible for the overexpression of *CtBPs* and the activation of CtBP-dependent downstream signaling.

## Discussion

**Figure 8:**
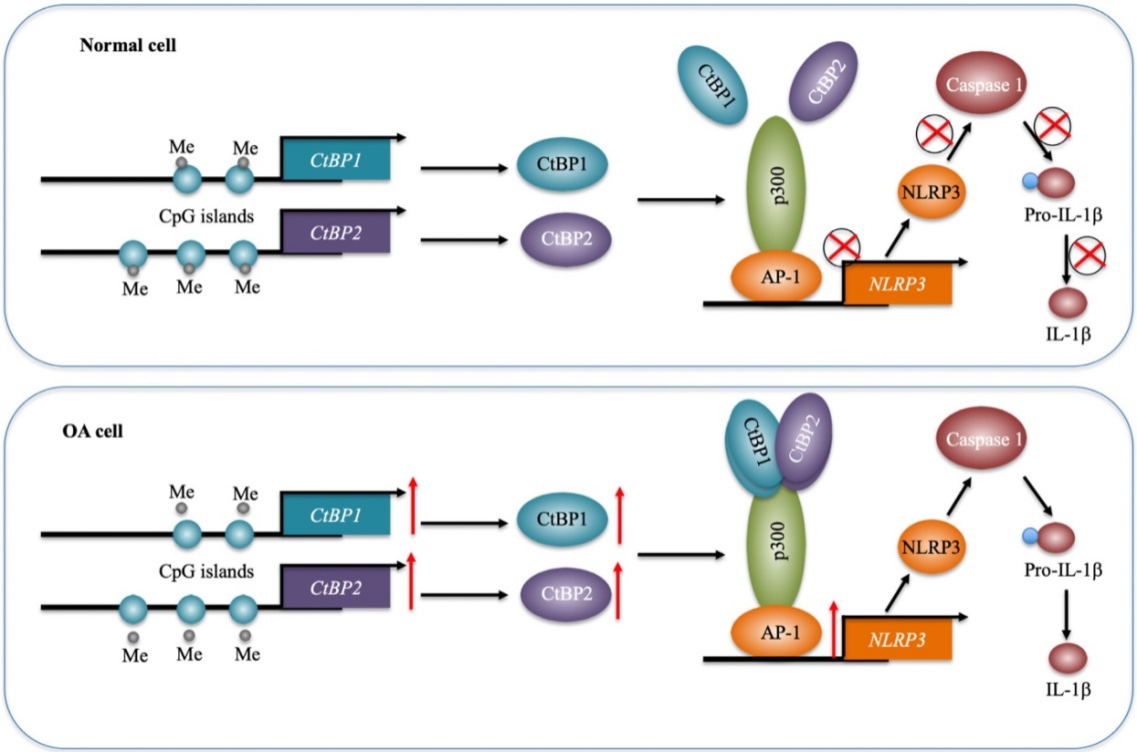
** A schematic model of the CPAC machinery in the pathogenesis of OA.** In normal cells, DNMTs maintain the DNA methylation of *CtBP* promoters at a basal level. In this state, the expression of CtBPs is very low. Thus, they cannot efficiently associate with p300 and AP1 subunits to assemble the CPAC machinery, and thus maintaining *NLRP3* expression at a basal level. In OA cells, the decreased DNA methylation levels in the promoters of *CtBPs* mediate their overexpression. Overexpressed CtBPs associate with p300 and AP1 subunits to assemble the CPAC machinery, which binds to the promoter *NLRP3* and activates its expression. The induced NLRP3 further activates Caspase-1 to cleave pro-IL-1β. The released IL-1β aggravates the inflammatory response and eventually contributes to the pathogenesis of OA.

Inflammation has been recently considered a critical factor that contributes to the occurrence of OA 1, 2. Although increased levels of proinflammatory cytokines such as TNF-α and IL-1β and activation of inflammasomes have been observed in OA for many years 5-10, the underlying mechanisms of how they are activated are still not fully understood. In the present study, we reveal a transcriptional regulatory mechanism of *NLRP3* in which downregulation of *DNMTs* causes a decrease in DNA methylation levels in the promoters of *CtBP1* and *CtBP2*, eventually leading to their overexpression. The overexpressed CtBPs form a transcriptional complex with p300 and AP1 subunits c-Jun and c-FOS, and this complex activates the expression of *NLRP3* through direct binding to its promoter. NLRP3 further activates Caspase-1 to cleave pro-IL-1β (Figure 8). Characterization of this complete signaling pathway provides important information for us to fully understand the pathogenesis of OA, and more importantly, our experimental results show that the blockage of CtBPs inhibits its downstream signaling, which provides an avenue for the therapy of OA.

Since their discovery, CtBPs have been known as transcriptional corepressors 32. Previous studies have indicated that CtBPs consistently function as corepressors in different cancers 32. In terms of molecular mechanisms, overexpressed CtBPs can form transcriptional complexes with numerous transcription factors to inhibit the expression of multiple tumor suppressor genes 32. However, it seems that CtBPs can function as either corepressors or coactivators in other diseases and biological processes 36-38. Biochemically, they also form a complex with HATs (or HDACs) and transcription factors to regulate gene transcription 28, 29, 32. In this study, we comprehensively verified the activation of *NLRP3* by the CPAC transcriptional machinery using a variety of experimental methods. To our knowledge, this is the first report to show that CtBPs can regulate the inflammatory response. Given the important role of inflammation in the pathogenesis of many diseases, we speculate that it would also have a similar activation role in the pathogenesis of other inflammation-related diseases. Although *CtBPs* are overexpressed in many cancers and other diseases, the underlying mechanism of their overexpression is still obscure. Some studies have revealed that miRNAs may be involved in the regulation of *CtBPs*. For example, Du and colleagues found that miR-485-3p negatively regulates the expression of *CtBP1* by directly binding to its 3'-untranslated region (3'-UTR) 46. Deng and colleagues identified that miR-137 can repress the expression of *CtBP1* by binding to its 3'-UTR 39. In this study, we identified that the overexpression of *CtBPs* is caused by the decreased DNA methylation levels in their promoters. This new mechanism also provides clues for revealing mechanisms of *CtBP* overexpression in other biological processes. Several small molecules, such as MTOB, NSC95397 and HIPP (hydroxyimino-3-phenylpropanoic acid), have been reported to exhibit strong and specific abilities to block CtBPs 32, which indicates that we can evaluate the role of these small molecules in the treatment of OA.

In addition to our study, many other studies have also identified that DNA methylation is involved in the pathogenesis of OA by affecting the expression of multiple genes, such as *COL2A1* (Collagen type II Alpha 1 chain), *COL9A1* (Collagen type IX Alpha 1 chain), *COL10A1* (Collagen type X Alpha 1 chain), *ACAN* (Aggrecan), *MMP3*, *MMP9*, *MMP13*, *ADAMTS4* (ADAM Metallopeptidase with thrombospondin type 1 motif) and *IL1B* 47, 48. In mammalian genomes, DNA methylation is mainly controlled by three DNMTs including DNMT1, DNMT3A and DNMT3B 49. Although many groups have reported the involvement of DNA methylation in the pathogenesis of OA 47, 48, most of them have not examined *DNMT* mRNA or protein levels in OA biopsies. Sesselmann and colleagues detected the expression of *DNMT1* and *DNMT3A* in OA chondrocytes but did not observe significant changes 50. Nakano and colleagues measured the protein levels of DNMT1 and DNMT3A in fibroblast-like synoviocytes (FLS) from rheumatoid arthritis (RA) and OA patients 51. Although they also did not find significant differences in these two proteins between RA and OA samples, they identified that IL-1β stimulation decreased *DNMT1* and *DNMT3A* mRNA levels in FLS 51. The reason for the difference between our study (~2-fold decrease in both *DNMT1* and *DNMT3A* mRNA levels) and other groups' results may be because of the sample population and controls. Sesselmann and colleagues only used 10 paired biopsies in their study 50. Nakano and colleagues compared DNMT1 and DNMT3A protein levels in RA-FLS and OA-FLS and did not use noninflammatory samples as controls 51. In contrast, we used 48 noninflammatory biopsies and 48 OA samples, which may be more reliable.

To identify CtBP-dependent genes, we conducted a microarray analysis and found multiple overlapping genes that were dependent on both *CtBP1* and *CtBP2*. Except for *NLRP3*, we also found other overlapping genes such as *IL-1B*, *IL-6*, *IL15*, *S100A8* (S100 calcium-binding protein A8), *S100A9*, *PTGS1* (Prostaglandin-endoperoxide synthase 1), *TNFA*, *VCAM1* (Vascular cell adhesion molecule 1), *TGFB1*, *CDH1*, *Bax*, *Bim* and *MMP13* (Supplementary Table-6 and -7). Interestingly, most of these genes including *IL-1B*, *IL-6*, *IL-15*, *PTGS1*, and *VCAM1* are NF-κB targets. The activation of NF-κB signaling is associated with inflammatory diseases, and it has also been reported that NF-κB signaling is involved in the pathogenesis of OA 23. Given that NF-κB is a transcription factor family, we speculate that CtBPs may also activate NF-κB-dependent signaling. We are currently investigating whether CtBPs can form a transcriptional complex with NF-κB. In our experiments, a very interesting phenomenon is that the protein levels of OA markers CD31, CD55 and CD68 are dependent on *CtBP* levels (Supplementary Figure 1). We speculate that one possibility is that these OA markers are downstream targets of *CtBPs*, and the other possibility may be because of the activation proinflammatory cytokines, which further activate OA markers. We will verify these two possibilities in the subsequent studies. The other interesting phenomenon is the mRNA level of *IL-1B* was dependent on *CtBPs* (Figure 2). The activation of NLRP3 only promotes the maturation of IL-1β instead of changing its mRNA level. Thus, we speculate that CtBPs may have different roles in the regulation of *IL-1B* mRNA and protein levels. As discussed above, there is a possibility that CtBPs form a complex with NF-κB to induce *IL-1B* mRNA level.

In summary, our results demonstrate that the DNA methylation mediates the expression of *CtBPs*. Overexpressed CtBPs form a transcriptional complex with p300 and AP1, whereby the CPAC transcriptional machinery specifically activates *NLRP3* expression and its downstream signaling. Our study found for the first time that CtBPs can regulate the inflammatory response. Our results provide new understanding and a breakthrough in the study of inflammation-related diseases.

## Supplementary Material

Supplementary figures and tables.
