# Supplementary material for: DNA methylation is involved in the pathogenesis of osteoarthritis by regulating CtBP expression and CtBP-mediated signaling
Source: Int J Biol Sci. 2020 Jan 30;16(6):994–1009. doi: 10.7150/ijbs.39945 (PMC7053340; doi:10.7150/ijbs.39945)
Supplement: Supplementary file 1 — Supplementary figures and tables. [file ijbsv16p0994s1.pdf]

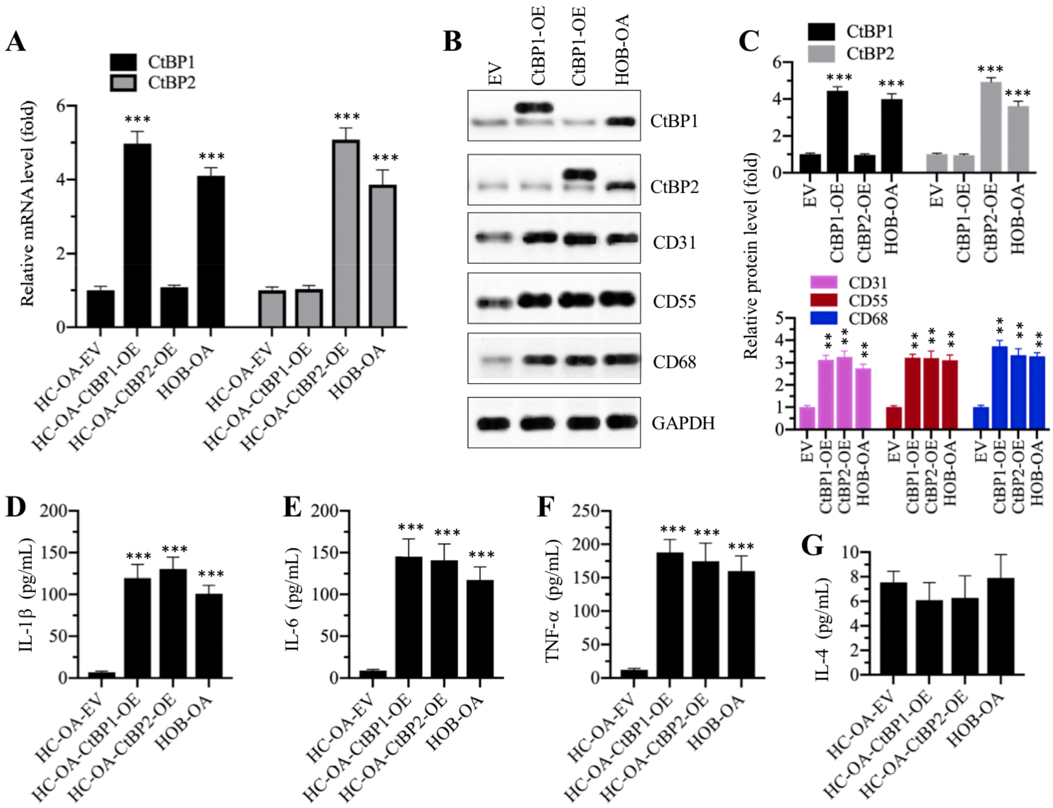

Supplementary Figure 1. Overexpression of *CtBPs* resulted in the activation of OA markers and the increase of proinflammatory cytokine concentrations.

(A) The mRNA levels of *CtBPs*. HC-OA cells were transfected with pCDNA3-2×Flag (empty vector, EV), pCDNA3-2×Flag-CtBP1 and pCDNA3-2×Flag-CtBP2, respectively. The resulting cells were used to measure mRNA levels of *CtBP1* and *CtBP2*. ***P<0.001. **(B)** Overexpression of *CtBPs* activated OA markers. Cells used in (A) were subjected to examine protein levels of CtBP1, CtBP2, CD31, CD55 and CD68. GAPDH was used as a loading control. **(C)** The quantified protein levels of CtBPs and OA markers. The intensity of protein bands in (B) was quantified using Image J software. **P<0.01 and ***P<0.001. **(D-G)** The concentrations of cytokines. Cells used in (A) were cultured for 48 h, and the supernatant of cell culture was used to

measure the concentrations of secreted cytokines including IL-1β **(D)**, IL-6 **(E)**, TNF-α **(F)**, and IL-4 **(G)** by ELISA assays. ****P*<0.001.

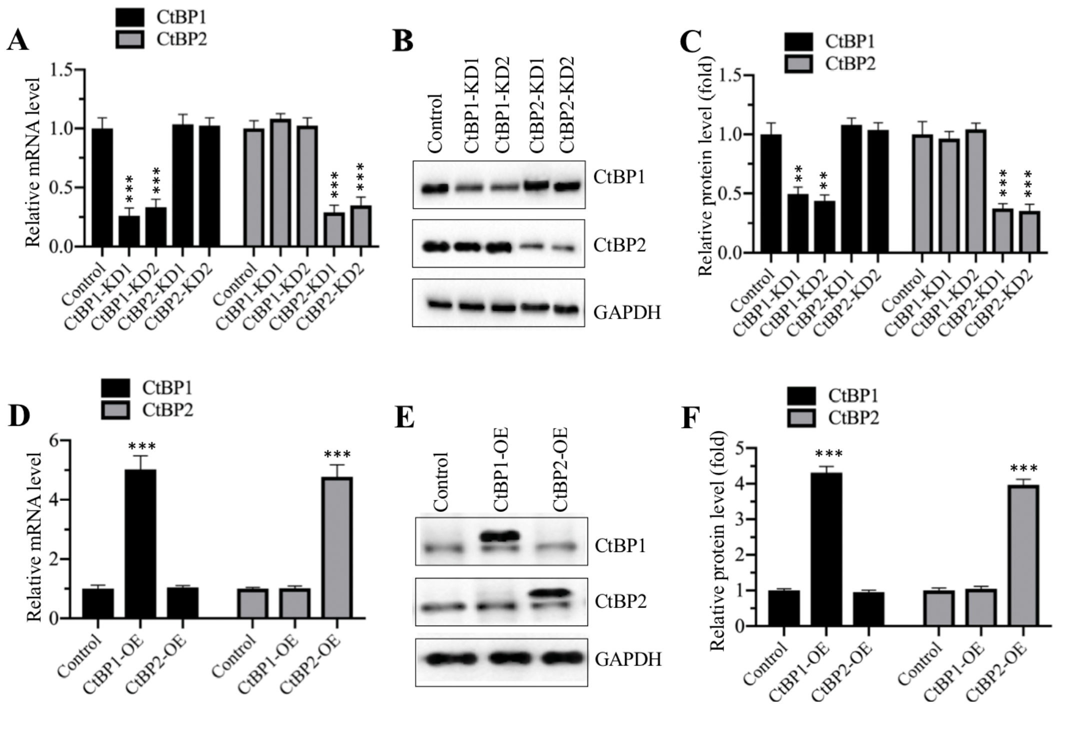

Supplementary Figure 2. CtBP mRNA and protein levels in CtBP-KD and CtBP-OE cells.

(A) The mRNA levels of *CtBPs* in CtBP-KD cells. HC-OA cells were transfected with siControl and two different siRNAs of *CtBP1* and *CtBP2* to generate Control, CtBP1-KD1, CtBP1-KD2, CtBP2-KD1, and CtBP2-KD2 cells, respectively. The resulting cells were used to measure the mRNA levels of *CtBP1* and *CtBP2.* ****P*<0.001. **(B)** The protein levels of CtBPs in CtBP-KD cells. Cells used in (A) were subjected to determine the protein levels of CtBP1 and CtBP2. GAPDH was used as a loading control. **(C)** The relative protein levels of CtBPs in CtBP-KD cells. The intensity of protein bands in (B) was quantified using Image J software. ***P*<0.01 and ****P*<0.001. **(D)** The mRNA levels of *CtBPs* in CtBP-OE cells. HC-OA cells were transfected with pCDNA3-2×Flag, pCDNA3-2×Flag-CtBP1 and

pCDNA3-2×Flag-CtBP2 to generate Control, CtBP1-OE, and CtBP2-OE cells, respectively. The resulting cells were used to measure the mRNA levels of *CtBP1* and *CtBP2*. ***P<0.001. **(E)** The protein levels of CtBPs in CtBP-OE cells. Cells used in (D) were subjected to determine the protein levels of CtBP1 and CtBP2. GAPDH was used as a loading control. **(F)** The relative protein levels of CtBPs in CtBP-OE cells. The intensity of protein bands in (E) was quantified using Image J software. ***P<0.001.

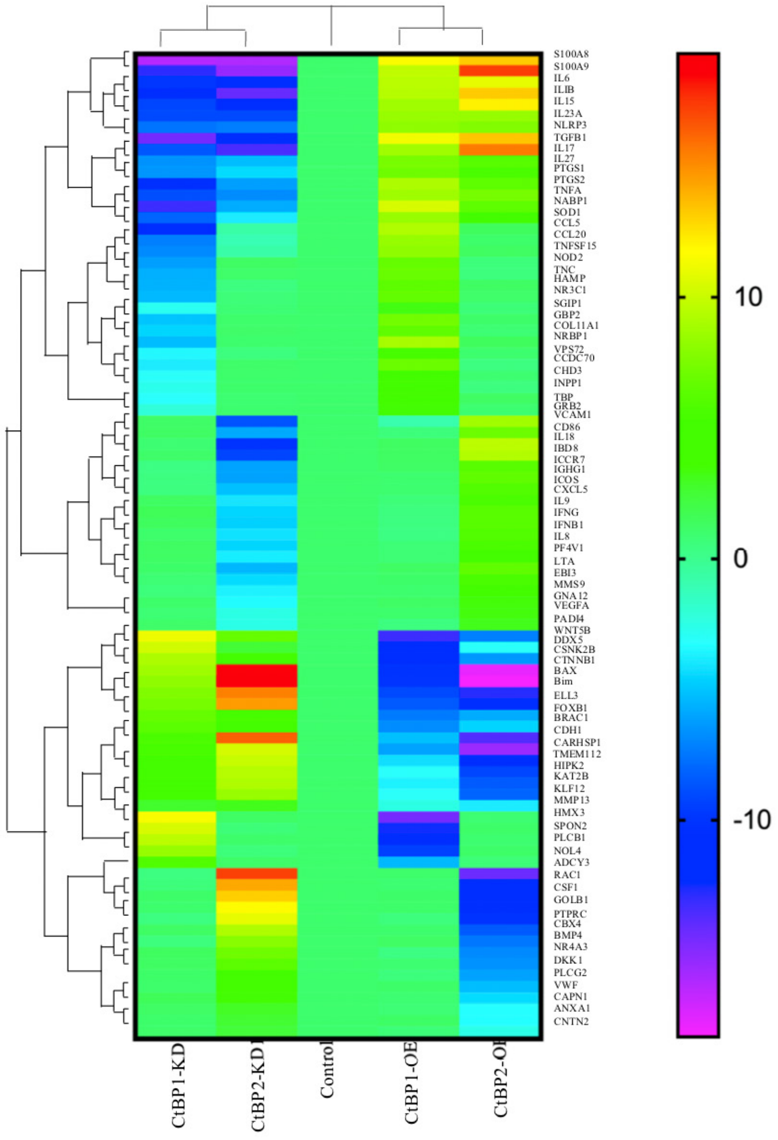

Supplementary Figure 3. The heatmap of differentially expressed genes dependent on *CtBP1* and *CtBP2*.

Total RNA from HC-OA (control), CtBP1-KD1, CtBP2-KD1, CtBP1-OE and CtBP2-OE cells were subjected to a microarray analysis. Genes regulated by *CtBP1* and *CtBP2* were shown.

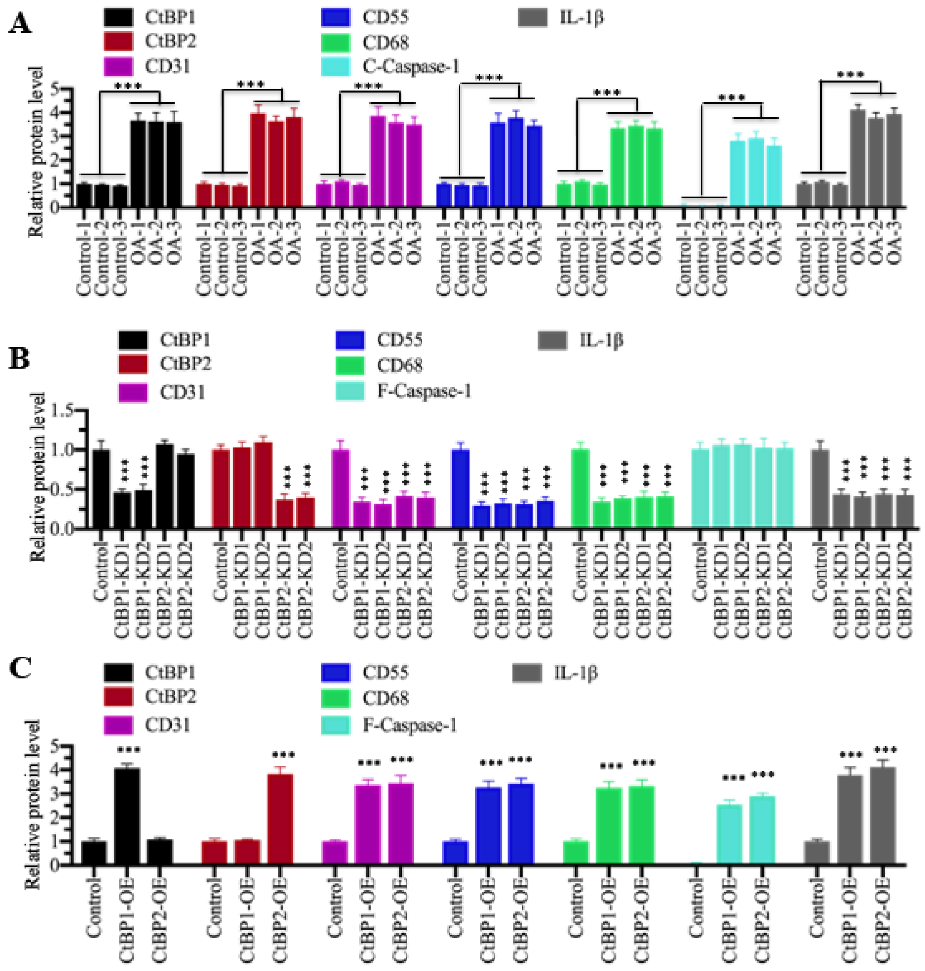

Supplementary Figure 4. The relative protein levels of OA markers and CtBP-downstream molecules in OA biopsies, CtBP-KD and CtBP-OE cells.

(A) The relative protein levels of OA markers and CtBP-downstream molecules in OA biopsies. The intensity of protein bands in Figure 3B was quantified using Image J software. ****P*<0.001. **(B)** The relative protein levels of OA markers and CtBP-downstream molecules in CtBP-KD cells. The intensity of protein bands in Figure 3C was quantified using Image J software. ****P*<0.001. **(C)** The relative protein levels of OA markers and CtBP-downstream molecules in CtBP-OE cells. The intensity of protein bands in Figure 3D was quantified using Image J software. ****P*<0.001.

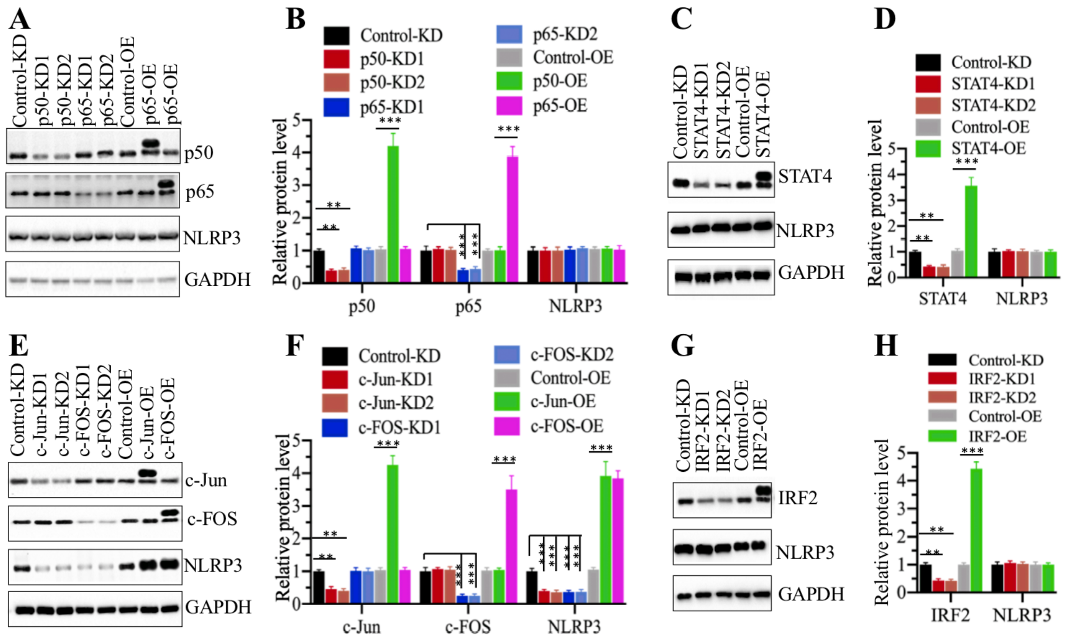

Supplementary Figure 5. The protein levels of transcription factors in their corresponding knockdown and overexpression cells.

(A) The protein levels of p50, p65 and NLRP3. Total cell extracts from cells used in Figure 4B were subjected to immunoblots to examine protein levels of p50, p65 and NLRP3. GAPDH was used as a loading control. **(B)** The relative protein levels of p50, p65 and NLRP3. The intensity of protein bands in (A) was quantified using Image J software. ***P*<0.01 and ****P*<0.001. **(C)** The protein levels of STAT4 and NLRP3. Total cell extracts from cells used in Figure 4C were subjected to immunoblots to examine protein levels of STAT4 and NLRP3. GAPDH was used as a loading control. **(D)** The relative protein levels of STAT4 and NLRP3. The intensity of protein bands in (C) was quantified using Image J software. ***P*<0.01 and ****P*<0.001. **(E)** The protein levels of c-Jun, c-FOS and NLRP3. Total cell extracts from cells used in Figure 4D were subjected to immunoblots to examine protein levels of c-Jun, c-FOS and NLRP3.

GAPDH was used as a loading control. **(F)** The relative protein levels of c-Jun, c-FOS and NLRP3. The intensity of protein bands in (E) was quantified using Image J software. **$P<0.01$ and ***$P<0.001$. **(G)** The protein levels of IRF2 and NLRP3. Total cell extracts from cells used in Figure 4E were subjected to immunoblots to examine protein levels of IRF2 and NLRP3. GAPDH was used as a loading control. **(H)** The relative protein levels of IRF2 and NLRP3. The intensity of protein bands in (G) was quantified using Image J software. **$P<0.01$ and ***$P<0.001$.

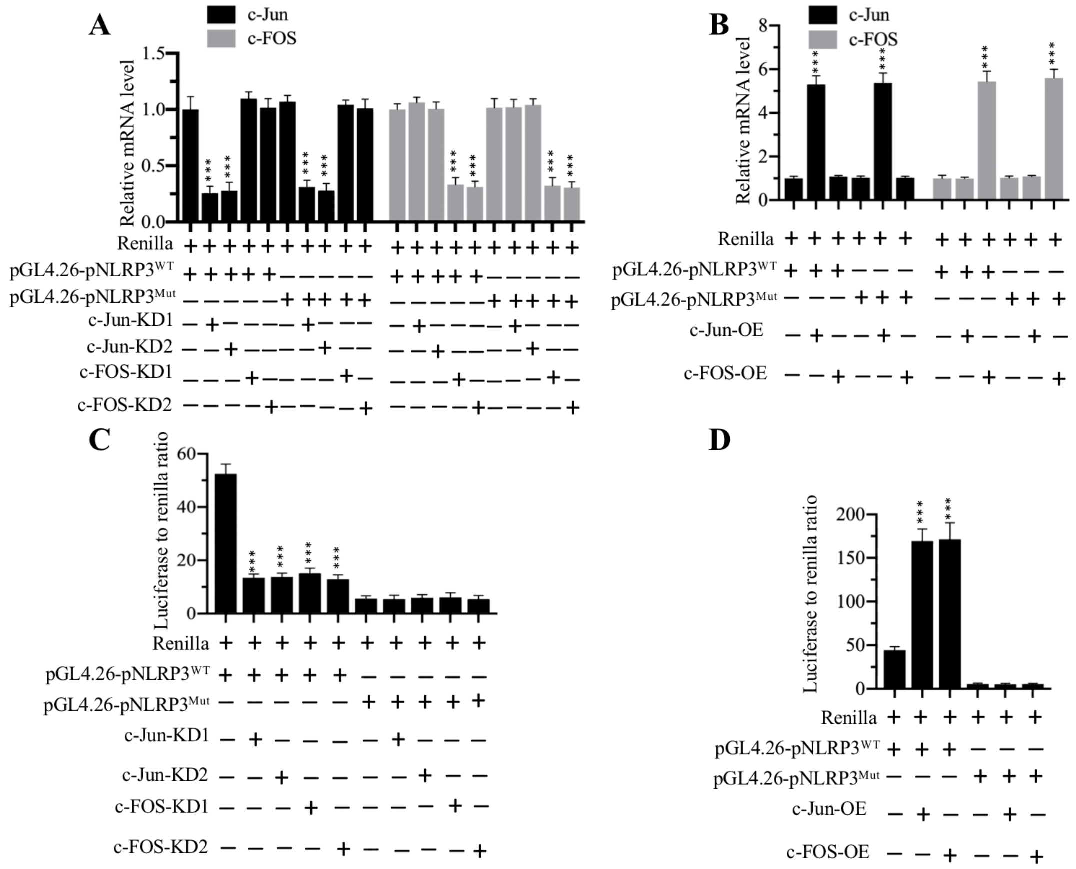

Supplementary Figure 6. Knockdown or overexpression of *AP1* subunits changed the luciferase activities mediated by *NLPR3* promoter.

(A and **B)** The relative mRNA levels of *c-Jun* and *c-FOS*. Different combinations of plasmids including pGL4.26-pNLRP3 + pRL-TK-Renilla and pGL4.26-pNLRP3^{Mut} + pRL-TK-Renilla plasmids were transfected into Control-KD, c-Jun-KD1, c-Jun-KD2, c-FOS-KD1, c-FOS-KD2, Control-OE, c-Jun-OE and c-FOS-OE cells, respectively. After culturing at 37°C for 48 h, cells were applied to RNA extraction and qRT-PCR analyses to examine the mRNA levels of *c-Jun* and *c-FOS*. ****P*<0.001. **(C** and **D)** The luciferase activities. The transfected cells used in (A and B) were applied to luciferase assays. ****P*<0.001.

c-Jun sequence

```
MTAKMETTFYDDALNASFLPSESGPYGYSNPKILKQSMTLNLADPVGSLKPHLRAKNSDL
LTSPDVGLLKLASPELERLIIQSSNGHITTTPTPTQFLCPKNVTDEQEGFAEGFVRALAE
LHSQNTLPSVTSAAQPVNGAGMVAPAVASVAGGSGSGGFSASLHSEPPVYANLSNFNPGA
LSSGGGAPSYGAAGLAFPAQPQQQQQPPHHLPQQMPVQHPRLQALKEEPQTVPEMPGETP
PLSPIDMESQERIKAERKRMRNRIAASKCRKRKLERIARLEEKVKTLKAQNSELASTANM
LREQVAQLKQKVMNHVNSGCQLMLTQQLQTF
```

c-FOS sequence

```
MMFSGFNADYEASSSRCSSASPAGDSLSYYHSPADSFSSMGSPVNAQDFCTDLAVSSANF
IPTVTAISTSPDLQWLVQPALVSSVAPSQTRAPHPFGVPAPSAGAYSRAGVVKTMTGGRA
QSIGRRGKVEQLSPEEEEKRRIRRERNKMAAAKCRNRRRELTDTLQAETDQLEDEKSALQ
TEIANLLKEKEKLEFILAAHRPACKIPDDLGFPEEMSVASLDLTGGLPEVATPESEEAFT
LPLLNDPEPKPSVEPVKSISSMELKTEPFDDFLFPASSRPSGSETARSVPDMDLSGSFYA
ADWEPLHSGSLGMGPMATELEPLCTPVVTCTPSCTAYTSSFVFTYPEADSFPSCAAAHRK
GSSSNEPSSDSLSSPTLLAL
```

Supplementary Figure 7. Protein sequences of c-Jun and c-FOS.

The human c-Jun and c-FOS protein sequences are shown. No PXDLS motif was found.

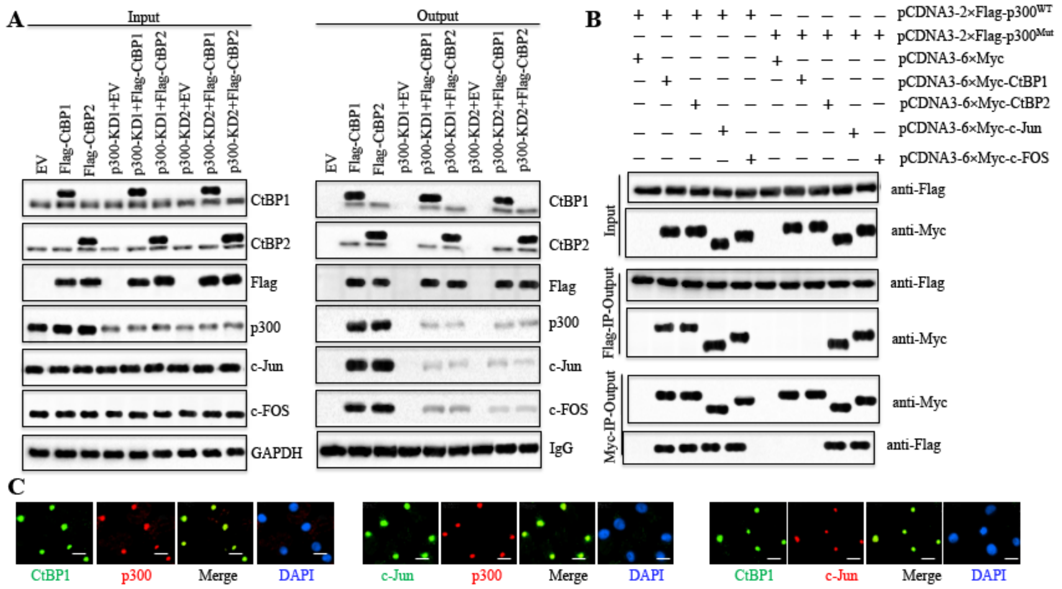

Supplementary Figure 8. Knockdown or mutation of p300 impaired the assembly of the CPAC and the colocalization of CPAC members in the nucleus.

(A) Knockdown of *p300* impaired the assembly of the CPAC. Different plasmids including pCDNA3-2×Flag (EV), pCDNA3-2×Flag-CtBP1, and pCDNA3-2×Flag-CtBP2 were transfected into HC-OA, p300-KD1 and p300-KD2 cells, respectively. After incubating for another 48 h, cells were lysed and 1/10 total cell extracts were taken out as input, and the other 9/10 cell extracts were subjected to IP analysis with an anti-Flag agarose. The input and output proteins were probed with anti-CtBP1, anti-CtBP2, anti-Flag, anti-p300, anti-c-Jun, anti-c-FOS antibodies, respectively. GAPDH and IgG were used as the control of input and output, respectively. **(B)** CtBPs interacted with p300 through the PMDLS motif. Different combinations of plasmids including pCDNA3-2×Flag-p300^{WT} + pCDNA3-6×Myc, pCDNA3-2×Flag-p300^{WT} + pCDNA3-6×Myc-CtBP1, pCDNA3-2×Flag-p300^{WT} + pCDNA3-6×Myc-CtBP2, pCDNA3-2×Flag-p300^{WT} + pCDNA3-6×Myc-c-Jun, pCDNA3-2×Flag-p300^{WT} + pCDNA3-6×Myc-c-FOS, pCDNA3-2×Flag-p300^{Mut} + pCDNA3-6×Myc, pCDNA3-2×Flag-p300^{Mut} + pCDNA3-6×Myc-CtBP1, pCDNA3-2×Flag-p300^{Mut} + pCDNA3-6×Myc-CtBP2, pCDNA3-2×Flag-p300^{Mut} + pCDNA3-6×Myc-c-Jun, pCDNA3-2×Flag-p300^{Mut} + pCDNA3-6×Myc-c-FOS were cotransfected into HC-OA cells. After incubating for another 48 h, cells were lysed and 1/10 total cell extracts were taken out as input, and the other 9/10 cell extracts were subjected to IP analysis with an anti-Flag agarose and anti-Myc agarose, respectively. The input and output proteins were probed with anti-Flag and anti-Myc antibodies,

respectively. **(C)** The colocalization of CPAC members. HOB-OA cells were stained with anti-CtBP1, anti-p300, and anti-c-Jun antibodies as indicated. The nuclei were stained with DAPI. Bars=100 μm.

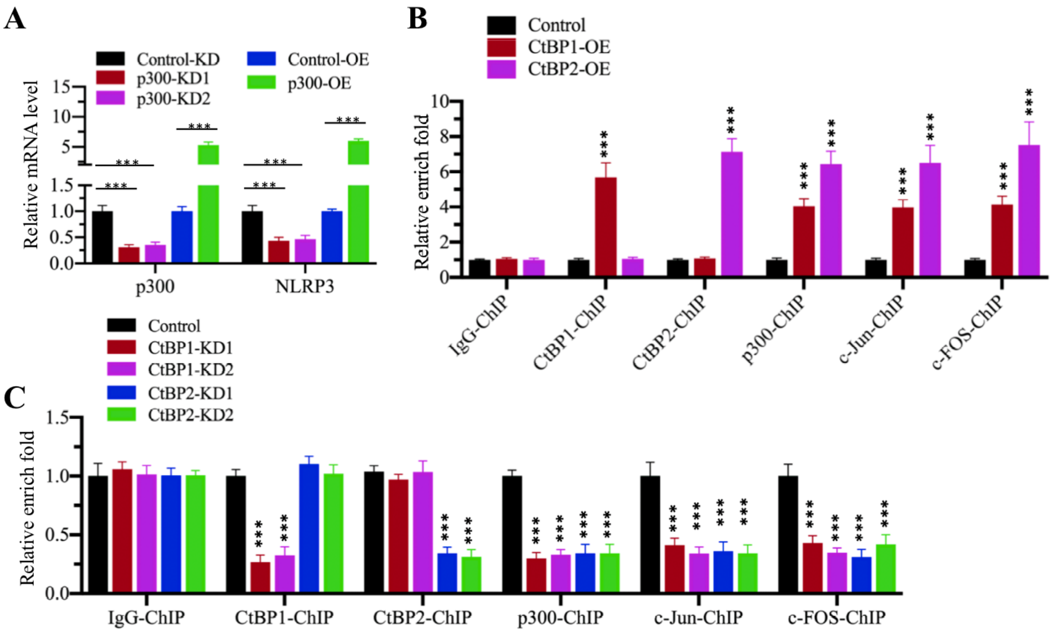

Supplementary Figure 9. The effects of *p300* knockdown and overexpression on *NLRP3* expression and the enrichment of CPAC members in the promoter of *NLRP3* in CtBP-KD and CtBP-OE cells.

(A) Knockdown or overexpression of *p300* changed the expression of *NLRP3*. HC-OA cells were transfected with siControl, two different siRNAs of p300, pCDNA3-2×Flag, and pcDNA3-2×Flag-p300 to generate the Control-KD, p300-KD1, p300-KD2, Control-OE, and p300-OE cells, respectively. Cells were used to examine the mRNA levels of *p300* and *NLRP3*. **(B)** The relative enrichment of CPAC members in CtBP-OE cells. The pCDNA3-2×Flag, pcDNA3-2×Flag-CtBP1, and pcDNA3-2×Flag-CtBP2 plasmids were transfected into HC-OA cells to generate Control-OE, CtBP1-OE, and CtBP2-OE cells, respectively. Cells were used to perform ChIP assays with IgG, anti-CtBP1, anti-CtBP2, anti-p300, anti-c-Jun, and anti-c-FOS antibodies, respectively. The enrichment of CPAC members in Control-OE was defined as one-fold. ***P<0.001. **(C)** The relative enrichment of CPAC members in CtBP-KD cells. The Control-KD, CtBP1-KD1, CtBP1-KD2, CtBP2-KD1, and CtBP2-KD2 cells were used to perform ChIP assays with IgG, anti-CtBP1, anti-CtBP2, anti-p300, anti-c-Jun, and anti-c-FOS antibodies, respectively. The enrichment of CPAC members in Control-KD was defined as one-fold. ***P<0.001.

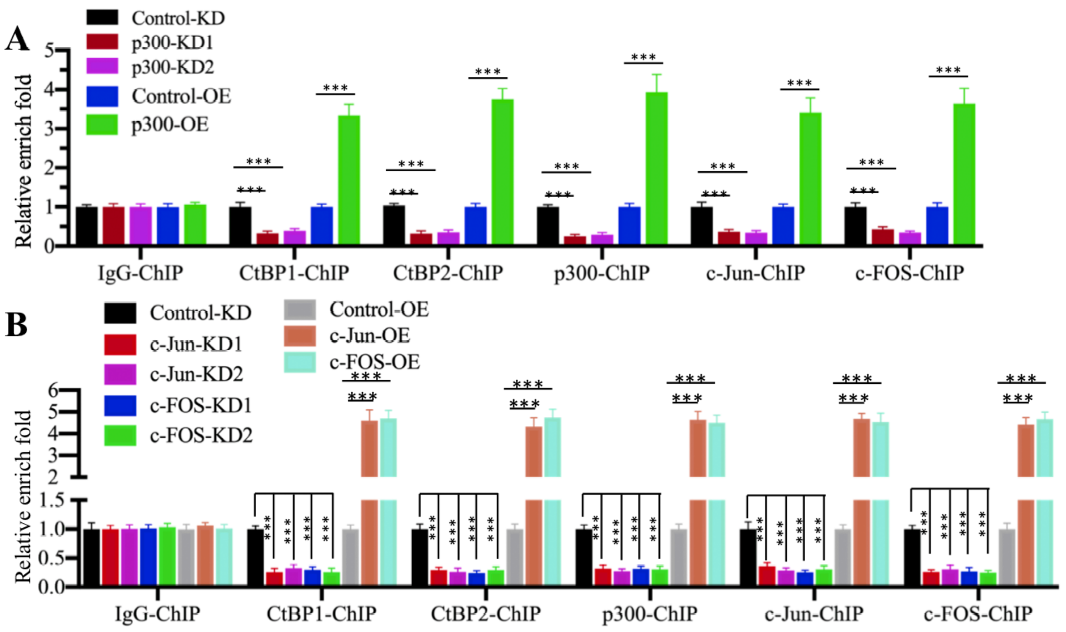

Supplementary Figure 10. Knockdown or overexpression of *p300* and *AP1* subunits adfected the binding of CPAC in the promoter of *NLRP3*.

(A) Knockdown or overexpression of *p300* affected the binding of CPAC in the promoter of *NLRP3*. The Control-KD, p300-KD1, p300-KD2, Control-OE, and p300-OE cells were used to perform ChIP assays with IgG, anti-CtBP1, anti-CtBP2, anti-p300, anti-c-Jun, and anti-c-FOS antibodies, respectively. The enrichment of CPAC members in Controls was defined as one-fold. ****P*<0.001. **(B)** Knockdown or overexpression of *c-Jun* and *c-FOS* affected the binding of CPAC in the promoter of *NLRP3*. The Control-KD, c-Jun-KD1, c-Jun-KD2, c-FOS-KD1, c-FOS-KD2, c-Jun-OE, and c-FOS-OE cells were used to perform ChIP assays with IgG, anti-CtBP1, anti-CtBP2, anti-p300, anti-c-Jun, and anti-c-FOS antibodies, respectively. The enrichment of CPAC members in Controls was defined as one-fold. ****P*<0.001.

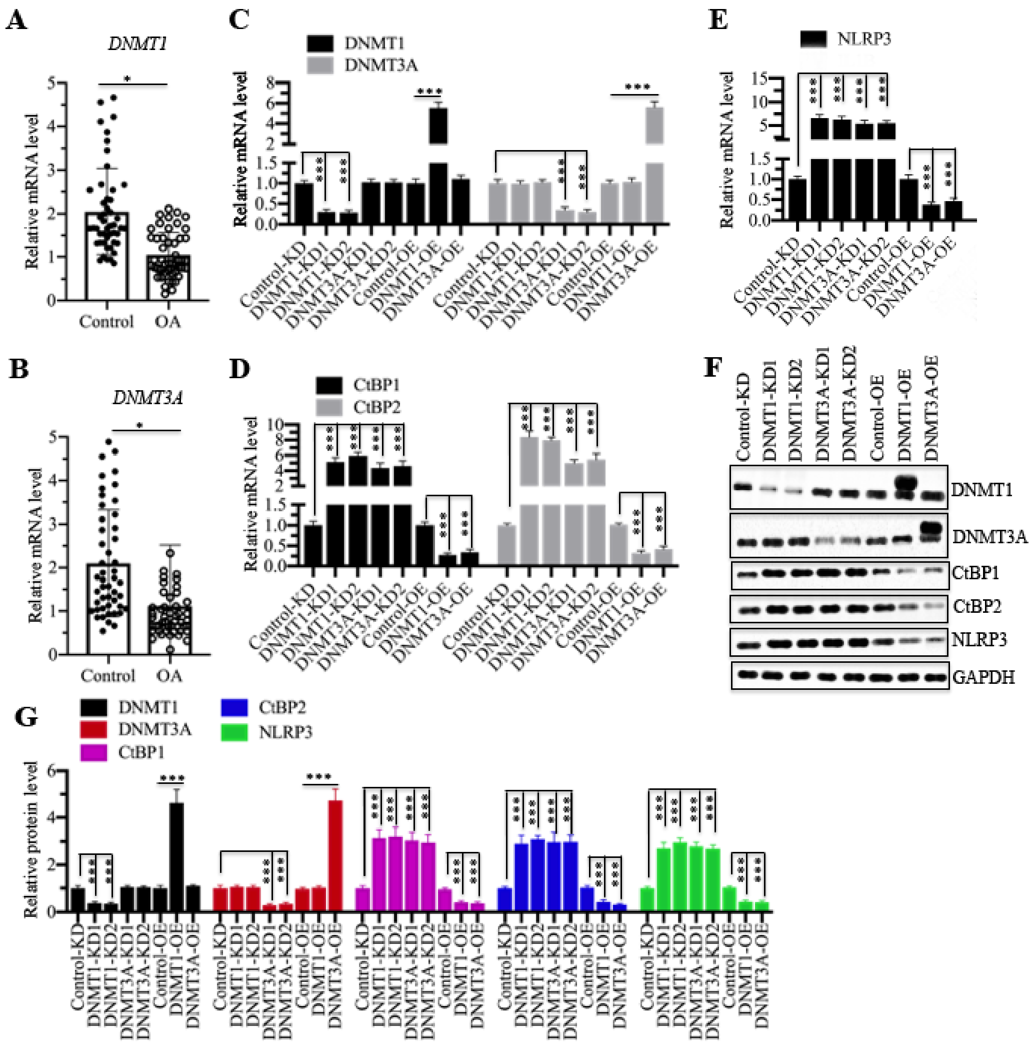

Supplementary Figure 11. The effects of DNMTs on the expression of *CtBPs* and their downstream molecules.

(A and B) Both *DNMT1* and *DNMT3A* mRNA levels were increased in OA biopsies. The mRNA levels of *DNMT1* **(A)** and *DNMT3A* **(B)** were measured in 48-paired biopsies from OA patients and controls by qRT-PCR analyses. The expression of *DNMT1* and *DNMT3A* in a healthy control was defined as one-fold. *P<0.05. **(C)** The mRNA levels of *DNMT1* and *DNMT3A* in their corresponding knockdown and overexpression cell lines. HC-OA cells were transfected with siControl, two different siRNAs of *DNMT1* and *DNMT3A*, pCDNA3-2×Flag, pcDNA3-2×Flag-DNMT1, and pcDNA3-2×Flag-DNMT3A to generate Control-KD, DNMT1-KD1, DNMT1-KD2, DNMT3A-KD1, DNMT3A-KD2, Control-OE, DNMT1-OE and DNMT3A-OE cells, respectively. The resulting cells were subjected to RNA isolation and qRT-PCR analyses to measure mRNA levels of *DNMT1* and *DNMT3A*. ***P<0.001. **(D and E)** The mRNA levels of *CtBPs* and *NLRP3* in DNMT-KD and DNMT-OE cells. Total

RNA samples used in (C) were used to examine mRNA levels of *CtBPs* **(D)** and *NLRP3* **(E)**. ***P<0.001. **(F)** The protein levels of DNMTs, CtBPs and NLRP3 in DNMT-KD and DNMT-OE cells. Cells used in (C) were subjected to determine protein levels of DNMT1, DNMT3A, CtBP1, CtBP2, and NLRP3. GAPDH was used as a loading control. **(G)** The relative protein levels of DNMTs, CtBPs and NLRP3 in DNMT-KD and DNMT-OE cells. The intensity of protein bands in (F) was quantified using the Image J software. ***P<0.001.

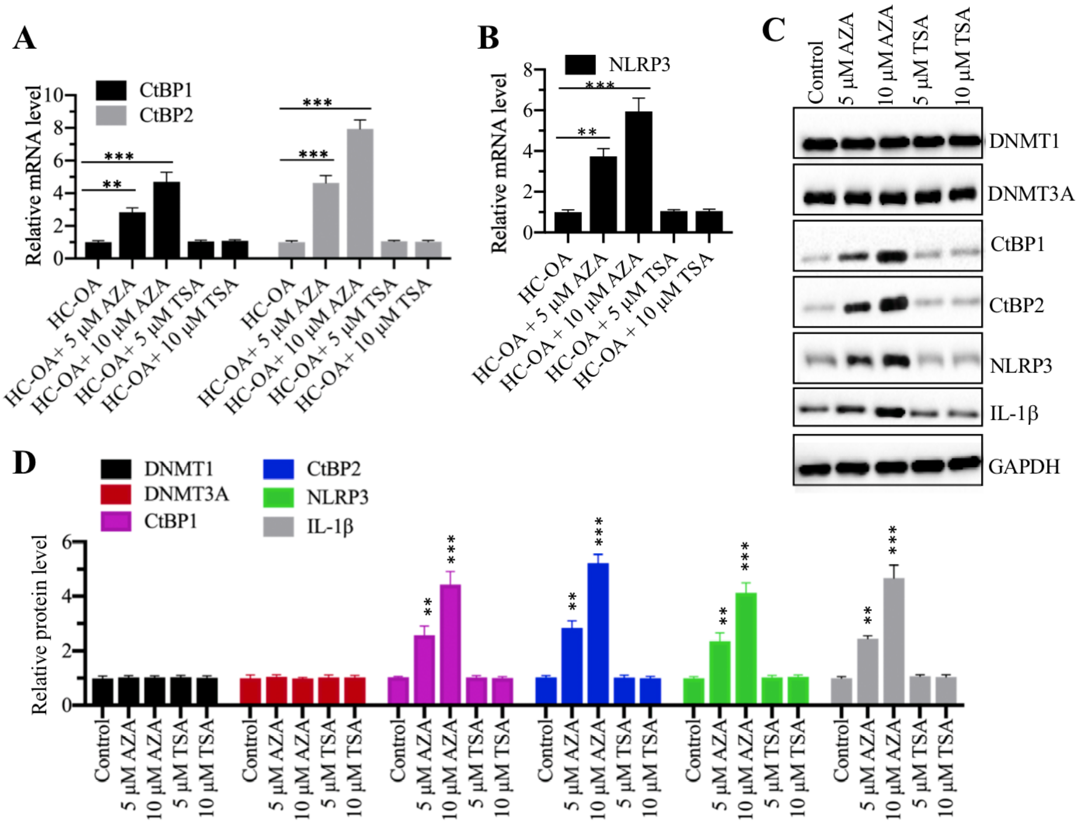

Supplementary Figure 12. AZA treatments induced CtBPs and their downstream molecules

(A) AZA treatments induced the mRNA levels of *CtBPs*. HC-OA cells were treated with 0, 5 and 10 μM AZA and TSA for 12 h, respectively. The treated cells were subjected to RNA isolation and qRT-PCR analyses to measure mRNA levels of *CtBP1* and *CtBP2*. ***P*<0.01 and ****P*<0.001. **(B)** AZA treatments induced the mRNA level of *NLRP3*. RNA samples used in (A) were applied to qRT-PCR analysis to examine mRNA level of *NLRP3*. ***P*<0.01 and ****P*<0.001. **(C)** The effects of AZA treatments on DNMTs, CtBPs, NLRP3 and IL-1β protein levels. Cells used in (A) were subjected to examine protein levels of DNMT1, DNMT3A, CtBP1, CtBP2, NLRP3 and IL-1β. GAPDH was used as a loading control. **(D)** The relative protein levels. The intensity of protein bands in (C) was quantified using Image J software. ***P*<0.01 and ****P*<0.001.

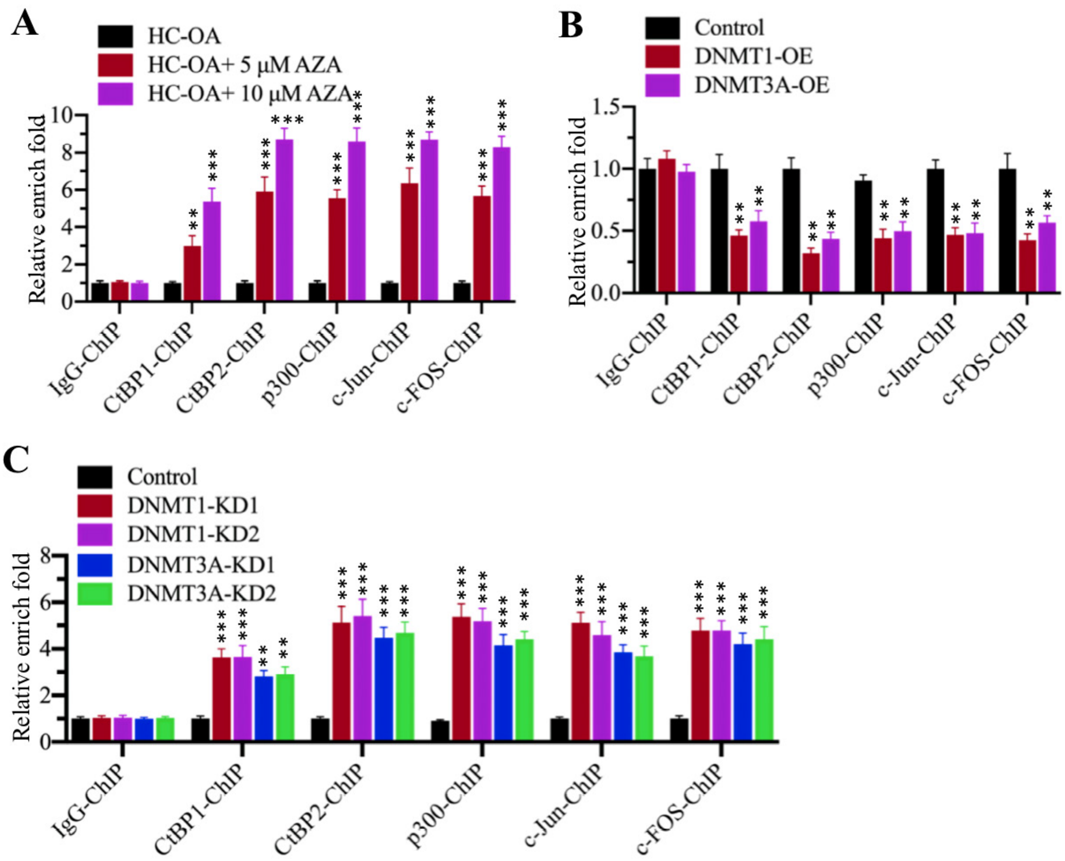

Supplementary Figure 13. The effects of AZA treatment and overexpression or knockdown of *DNMTs* on the enrichment of CPAC members in the promoter of *NLRP3*.

(A) AZA treatments increased the enrichment of CPAC members in the promoter of NLRP3. HC-OA cells were treated with 0, 5 and 10 μM AZA for 12 h. The treated cells were subjected to ChIP assays using IgG, anti-CtBP1, anti-CtBP2, anti-p300, anti-c-Jun and anti-c-FOS antibodies and qRT-PCR analyses to measure their enrichment in the promoter of *NLRP3*. $^{**}P<0.01$ and $^{***}P<0.001$. **(B)** Overexpression of *DNMTs* decreased the enrichment of CPAC members in the promoter of *NLRP3*. The Control-OE, DNMT1-OE, and DNMT3A-OE cells were subjected to ChIP assays using IgG, anti-CtBP1, anti-CtBP2, anti-p300, anti-c-Jun and anti-c-FOS antibodies and qRT-PCR analyses to measure their enrichment in the promoter of *NLRP3*. $^{**}P<0.01$. **(C)** Knockdown of *DNMTs* decreased the enrichment of CPAC members in the promoter of *NLRP3*. The Control-KD, DNMT1-KD1, DNMT1-KD2, DNMT3A-KD1 and DNMT3A-KD2 cells were subjected to ChIP assays using IgG, anti-CtBP1, anti-CtBP2, anti-p300, anti-c-Jun and anti-c-FOS antibodies and qRT-PCR analyses to measure their enrichment in the promoter of *NLRP3*. $^{**}P<0.01$

and ***P<0.001. The enrichment of CPAC members in Controls was defined as one-fold.

Supplementary Table-1. The basic information of controls and OA patients

Parameter	Controls (n=48)	OA (n=48)
Mean age	35.8±4.2	55.4±6.3
Gender	34M/14F	20M/28F
Severe stage	N/A	**3**

F, female; M, male. N/A, not available.

Supplementary Table-2. Primers used in qRT-PCR assays

Gene	Forward	Reverse
CtBP1	TCTCACCAGGGAGGACCTGGA	CTGCTCGACGCTCTGGACTCGT
CtBP2	AGATCATGAACGGCCCCCTGC	GGTGATGGTGTGGTACATCATGT
NLRP3	ATCCCACTGTGATATGCCAGG	CCCAGACGGGCATTCCTG
IL-1B	CACTACAGCAAGGGCTTCAGG	GTTCAGTGATCGTACAGGTGC
S100A8	ACTCTATCATCGACGTCTACCA	CCTGATATACTGAGGACACTC
Bax	GACAGTAACATGGAGCTG	GAAAAGGGCGACAACCCGG
Bim	AGGCAGGCTGAACCTGCAGATA	TGGGTGGTCTTCGGCTGCT
CDH1	ACCCTGGCTTTGACGCCGAG	TCACACCATCTGTGCCCACT
p65	CTCTGGCAGCTGCCTCGGTG	CCGCAGCTGCATGGAGAC
p50	ACTGTGAGGATGGGATCTG	TACACGCCTCTGTCATTCG
c-Jun	AACTCGGACCTCCTCACCT	CGCACGAAGCCCTCGGCGAA
c-FOS	GACCTGCAGTGGCTGGTGCAG	CTGTTCCACCTTGCCCCTCCT
STAT4	CGGCATCTGTTGGCCCAATGG	GATTGTGTATCAAGAGTAGGT
IRF2	GGATGCATGCGGCTAGACAT	TGGCGCATCTGAAATTCGCCT
p300	ACCTTCCCCACTGTCGCACAA	GGGGAGACACACAGGACAAT
DNMT1	GAAGCCCGTAGAGTGGGAA	GATGTGATGGTGGTTTGCCTG
DNMT3a	TGGCAAGGAGGAGCGCCAAG	GGTAATAGCTCTGAGGCGCCT
Actin	CACCAACTGGGACGACAT	ACAGCCTGGATAGCAACG

Supplementary Table-3. Primers used vector constructions

Gene	Forward	Reverse
CtBP1	CGGGATCCATGGGCAGCTCGCACTTGCTCA	CGGAATTCCTACAACTGGTCACTGGCGTGGT
CtBP2	CGGGATCCATGGCCCTTGTGGATAAGCACAA	CGGAATTCCTATTGCTCGTTGGGGTGCTCTCGA
p65	CGGGATCCATGGACGAACTGTTCCCCCTC	CGGAATTCTGCTGAGTCAGATCAGCTCCTAA
p50	CGGGATCCATGGCAGAAGATGATCCATATT	CGGAATTCCTAAATTTTGCCTTCTAGAGGTC
STAT4	CGGGATCCATGTCTCAGTGGAATCAAGTC	CGGAATTCTCATTCAGCAGAATAAGGAGACTT
c-Jun	CGGGATCCATGACTGCAAAGATGGAAACG	CGGAATTCTCAAAATGTTTGCAACTGCTGC
c-FOS	CGGGATCCATGATGTTCTCGGGCTTCAACG	CGGAATTCTCACAGGGCCAGCAGCGTGGGTGA
IRF2	CGGGATCCATGCCGGTGGAAAGGATGCGC	CGGAATTCTTAACAGCTCTTGACGCGGGCC
p300	CGGGATCCATGGCCGAGAATGTGGTGGAA	CGGAATTCCTAGTGTATGTCTAGTGTACTC
$p300^{Mut}$	CGGGATCCGTGAAGAGCCGCATGCGTCGTTCTA CCATTAAGA	CGGAATTCTCTTAATGGTAGAACGACGCATGCG GCTCTTCAC
DNMT 1	CGGGATCCATGCCGGCGCGTACCGCCCCA	CGGAATTCCTAGTCCTTAGCAGCTTCCTC
DNMT 3A	CGGGATCCATGCCCGCCATGCCCTCCAGCG	CGGAATTCTTACACACACGCAAAATACTCCTTC
NLRP3 promoter	CGGGGTACCCTTGCTCTTGTCACCCAGGCT	CCGCTCGAGAATGAATTTATAGCAGTCGCAGCC
$pNLRP3^{Mut}$	GAACAGGTCCAGCAATCCAGCAGGGAG	CTCCCTGCTGGATTGCTGGACCTGTTC

Supplementary Table-4. Primers used in ChIP-qRT-PCR assays

Promoter	Forward	Reverse
NLRP3	TCTCCTCAAGCTACTCAAGCTG	GGTTCTCTCCGACATGTTCTAC

Supplementary Table-5. Primers used in qMSP assays

Promoter	Forward	Reverse
CtBP1-CpG1	TTGGTTGAGGGTTTAGTATTGTTAG	AATAATTACATAATTTCAAAAACCAC
CtBP1-CpG2	AGTTTTTCGTTAGGTTTTCGTTTC	GATTAATCTCCTAATTCCCAACG
CtBP2-CpG1	GATTTTAATTTTGAGACGTTAGGAC	TTAAAAAACCCTATATTAAATCGAA
CtBP2-CpG2	GTATTAGGAGGAAGTTGGAGTTTG	AACAACCAACCACATAAAAAAACA
CtBP2-CpG3	GGAGTTATTAATTTTTCGAGAGAGTC	TAAACGAAAAACGAAATAAAAATCG

Supplementary Table-6. Differentially expressed genes dependent on *CtBP1*

Gene	Gene Description	Change fold in CtBP1-KD cells	Change fold in CtBP1-OE cells
S100A8	S100 calcium binding protein A8	-16.4	11.5
NLRP3	NLR family pyrin domain containing 3	-14.5	11.2
TNFA	Tumor necrosis factor alpha	-13.2	10.4
S100A9	S100 calcium binding protein A9	-12.9	9.7
IL-1B	Interleukin-1 beta	-11.7	9.4
SOD1	Superoxide dismutase 1	-11.2	9.2
PTGS1	Prostaglandin-endoperoxide synthase 1	-10.4	9.1
IL-6	Interleukin-6	-9.9	9.5
IL-15	Interleukin-15	-9.2	8.7
IL-23A	Interleukin-23A	-9.1	8.5
PTGS2	Prostaglandin-endoperoxide synthase 2	-8.9	8.7
TGFB1	Transforming growth factor beta 1	-8.6	8.7
NABP1	Nucleic acid binding protein 1	-8.1	8.5
ICAM1	Intercellular adhesion molecule 1	-7.6	8.1
CCL5	C-C motif chemokine ligand 5	-7.2	8.4
CCL20	C-C motif chemokine ligand 20	-6.9	8.1
IL-17	Interleukin-17	-6.6	7.4
IL-27	Interleukin-27	-6.5	7.1
TNFSF15	TNF superfamily member 15	-6.2	6.7
NOD2	Nucleotide binding oligomerization domain containing 2	-5.7	6.8
TNC	Tenascin C	-5.6	6.4
HAMP	Hepcidin antimicrobial peptide	-5.4	6.6
COL11A1	Collagen, type XI, alpha 1	-5.3	8.9
SGIP1	SH3 domain GRB2-like protein 3-interaction protein 1	-5.1	7.2
GBP2	Guanylate binding protein 2	-4.5	6.5
VPS72	Vacuolar protein sorting-associated protein 72	-3.9	6.9

NRBP1	Nuclear receptor-binding protein 1	3.5	5.4
INPP1	Inositol polyphosphate 1-phosphatase	-3.2	4.6
CCDC70	Coiled-coil domain-containing protein 70	-3.1	5.1
NR3C1	Nuclear receptor subfamily 3 group C member 1	-2.9	3.2
CHD3	Chromodomain helicase DNA binding protein 3	-2.7	4.4
TBP	TATA-Box binding protein	-2.2	3.5
GRB2	Growth factor receptor bound protein 2	11.4	-14.6
WNT5B	Wnt family member 5B	11.1	-13.2
PLCB1	Phospholipase C beta 1	10.4	-12.5
DDX5	DEAD-box helicase 5	10.2	-11.8
HMX3	H6 family homeobox 3	9.6	-11.1
CSNK2B	Casein Kinase 2 Beta	9.1	-11.8
CTNNB1	Catenin beta 1	8.7	-10.2
SPON2	Spondin 2	8.4	-9.4
Bax	BCL2 associated X protein	8.1	-10.1
Bim	BCL2 like 11	7.6	-9.1
ELL3	Elongation factor RNA polymerase II-like 3	7.3	-8.5
FOXB1	Forkhead box B1	6.7	-7.4
BRAC1	Breast cancer type 1 susceptibility protein 1	6.3	-6.8
NOL4	Nucleolar protein 4	5.8	-5.4
CDH1	Cadherin 1	5.7	-5.2
CARHSP1	Calcium-regulated heat stable protein 1	5.3	-6.1
TMEM112	Lipase maturation factor 1	5.1	-4.3
HIPK2	Homeodomain interacting protein kinase 2	4.5	-3.2
KAT2B	Lysine acetyltransferase 2B	4.1	-3.7
KLF12	Kruppel like factor 12	3.6	-3.1
MMP13	Matrix metallopeptidase 13	2.7	-2.5

Supplementary Table-7. Differentially expressed genes dependent on *CtBP2* (The same genes as Supplementary Table-5 were labeled with red color)

Gene	Gene Description	Change fold in CtBP1-KD cells	Change fold in CtBP1-OE cells
S100A9	S100 calcium binding protein A9	-16.2	17.3
S100A8	S100 calcium binding protein A8	-15.5	13.2
IL-6	Interleukin-6	-15.3	14.3
IL-1B	Interleukin-1 beta	-14.2	13.2
TGFB1	Transforming growth factor beta 1	-13.5	15.6
IL-15	Interleukin-15	-11.4	12.1
IL-18	Interleukin-15	-10.2	9.6
NLRP3	NLR family pyrin domain containing 3	-9.5	9.2
IBD8	Inflammatory bowel disease 8	-9.2	9.3
IL-23A	Interleukin-23A	-9.1	8.4
VCAM1	Vascular Cell Adhesion Molecule 1	-8.7	8.8
TGFB1	Transforming growth factor beta 1	-8.2	8.5
LTB	Lymphotoxin beta	-7.6	8.2
ICAM1	Intercellular adhesion molecule 1	-7.3	7.7
PTGS2	Prostaglandin-endoperoxide synthase 2	-6.8	7.3
PTGS1	Prostaglandin-endoperoxide synthase 1	-6.2	6.4
IGHG1	Immunoglobulin heavy constant gamma 1	-6.1	6.5
CCR7	C-C motif chemokine receptor 7	-6.1	6.2
CD86	CD86 antigen	-5.9	7.0
TNFA	Tumor necrosis factor alpha	-5.8	6.4
LTA	Lymphotoxin alpha	-5.5	6.5
IL-17	Interleukin-17	-5.3	6.1
ICOS	Inducible T cell Costimulator	-5.2	5.8
IFNG	Interferon gamma	-4.7	6.2
IL-9	Interleukin-9	-4.5	6.1
IL-8	Interleukin-8	-4.5	5.5
EBI3	Epstein-barr virus induced 3	-4.4	5.6
IL-27	Interleukin-27	-4.4	5.1

IFNB1	Interferon beta 1	-4.2	5.8
CXCL5	C-X-C motif chemokine ligand 5	-4.1	5.5
INPP1	Inositol polyphosphate 1-phosphatase	-4.0	5.1
NABP1	Nucleic acid binding protein 1	-3.9	4.6
PF4V1	Platelet factor 4 variant 1	-3.8	4.2
MMS9	Matrix metallopeptidase 9	-3.7	4.0
GNA12	G Protein subunit alpha I2	-3.4	3.4
PADI4	Peptidyl arginine deiminase 4	-2.6	3.1
VEGFA	Vascular endothelial growth factor A	-2.5	2.3
Bax	BCL2 associated X protein	19.3	-18.3
CTNNB1	Catenin beta 1	18.5	-17.8
ADCY3	Adenylate cyclase 3	17.2	-14.3
CDH1	Cadherin 1	16.3	-13.7
Bim	BCL2 like 11	15.4	-12.8
ELL3	Elongation factor RNA polymerase II-like 3	14.6	-12.1
RAC1	Rac family small GTPase 1	14.2	-11.7
CSF1	Colony stimulating factor 1	13.1	-11.3
GOLGB1	Golgin B1	11.6	-10.6
PTPRC	Protein tyrosine phosphatase receptor type C	10.9	-10.2
CARHSP1	Calcium-regulated heat stable protein 1	10.3	-15.6
TMEM11	Transmembrane protein 11	9.9	-11.2
HIPK2	Homeodomain interacting protein kinase 2	9.4	-9.5
KAT2B	Lysine acetyltransferase 2B	9.2	-8.4
KLF12	Kruppel like factor 12	8.5	-8.1
CBX4	Chromobox 4	7.9	-7.5
BMP4	Bone morphogenetic protein 4	7.1	-6.9
WNT5B	Wnt family member 5B	6.7	-7.2
NR4A3	Nuclear receptor subfamily 4 group A member 3	6.3	-6.6
FOXB1	Forkhead box B1	5.4	-5.8
CSNK2B	Casein Kinase 2 Beta	5.1	-6.4
DKK1	Dickkopf WNT signaling pathway inhibitor 1	4.6	-6.1

PLCG2	Phospholipase C gamma 2	4.5	-5.3
CARHSP1	Calcium-regulated heat stable protein 1	4.1	-5.1
BRAC1	Breast cancer type 1 susceptibility protein 1	3.7	-4.6
VWF	Von willebrand factor	3.5	-4.4
MMP13	Matrix metallopeptidase 13	3.1	-3.9
CAPN1	Calpain 1	2.9	-4.2
ANXA1	Annexin A1	2.6	-3.3
DDX5	DEAD-box helicase 5	2.5	-3.1
CNTN2	Contactin 2	2.4	-2.6
